# Novel Proteasome Inhibitors and Histone Deacetylase Inhibitors: Progress in Myeloma Therapeutics

**DOI:** 10.3390/ph10020040

**Published:** 2017-04-11

**Authors:** Saurabh Chhabra

**Affiliations:** Division of Hematology/Oncology, Department of Medicine, Medical College of Wisconsin, 9200 W Wisconsin Ave, Milwaukee, WI 53226, USA; schhabra@mcw.edu

**Keywords:** multiple myeloma, proteasome inhibitors, histone deacetylase inhibitors, clinical trials in myeloma

## Abstract

The unfolded protein response is responsible for the detection of misfolded proteins and the coordination of their disposal and is necessary to maintain the cellular homoeostasis. Multiple myeloma cells secrete large amounts of immunoglobulins, proteins that need to be correctly folded by the chaperone system. If this process fails, the misfolded proteins have to be eliminated by the two main garbage-disposal systems of the cell: proteasome and aggresome. The blockade of either of these systems will result in accumulation of immunoglobulins and other toxic proteins in the cytoplasm and cell death. The simultaneous inhibition of the proteasome, by proteasome inhibitors (PIs) and the aggresome, by histone deacetylase inhibitors (HDACi) results in a synergistic increase in cytotoxicity in myeloma cell lines. This review provides an overview of mechanisms of action of second-generation PIs and HDACi in multiple myeloma (MM), the clinical results currently observed with these agents and assesses the potential therapeutic impact of the different agents in the two classes. The second-generation PIs offer benefits in terms of increased efficacy, reduced neurotoxicity as off-target effect and may overcome resistance to bortezomib because of their different chemical structure, mechanism of action and biological properties. HDACi with anti-myeloma activity in clinical development discussed in this review include vorinostat, panobinostat and selective HDAC6 inhibitor, ricolinostat.

## 1. Introduction

The outcome of multiple myeloma (MM) patients has dramatically improved in recent years and this has been possible essentially due to the introduction of new classes of agents, namely proteasome inhibitors (PIs) and immunomodulatory drugs (IMIDs) [1]. Nevertheless, vast majority of MM patients experience a pattern of responses and relapses, with each relapse generally being of shorter duration than the previous ones. Therefore, there is a need for anti-myeloma therapies with novel mechanisms of action or therapy based on the mechanisms of action that have already been demonstrated to be effective [2]. Inhibition of proteasome, a proteolytic complex responsible for the degradation of ubiquitinated proteins, has emerged as a powerful treatment strategy. The first-in-class PI bortezomib (BTZ) has demonstrated the feasibility of this approach and validated the proteasome as a therapeutic target [3,4]. Preclinical studies suggested proteasome inhibition with BTZ resulted in pleiotropic effects, disrupting multiple cellular signaling pathways and inducing tumor cell death [4,5]. Nonetheless, some limitations of BTZ have become evident, including primary resistance in some patients with MM, acquisition of resistance in many who respond initially and development of treatment-emergent peripheral neuropathy (PN) [6,7,8,9,10,11,12,13]. To overcome the pitfalls of BTZ, several second-generation PIs have been developed with the aim of improving anti-tumor efficacy by increasing the potency of proteasome inhibition while trying to reduce toxicity by improving proteasome binding duration, thereby reducing “off-target” effects [14,15,16,17,18]. Several of these new agents: carfilzomib, ixazomib, marizomib and oprozomib are based on moieties different from BTZ and have modified pharmacologic properties, potentially resulting in improved efficacy and decreased neurotoxicity that is characteristic of BTZ (Table 1). Histone deacetylase inhibitors (HDACi) are a class of drugs that has emerged as a unique treatment option for MM due to the aberrant epigenetic gene expression patterns and overproduction of misfolded proteins commonly present in MM cells [19]. HDACi bind to the catalytic domains of histone deacetylases (HDACs), downregulating their activity and inhibit myeloma cell survival and proliferation [20]. Studies, both preclinical and clinical, have supported synergistic activity of HDACi and other anti-myeloma agents, most strikingly with PIs. This review summarizes the promising results seen in patients with MM with the newer PIs and HDACi (Table 2 and Table 3). With the discovery of new targets and rationally designing the clinical trials of the targeted combination therapies, the elimination of resistant myeloma clones may be possible [21].

## 2. Proteasome: A Therapeutic Target

**Figure 1:**
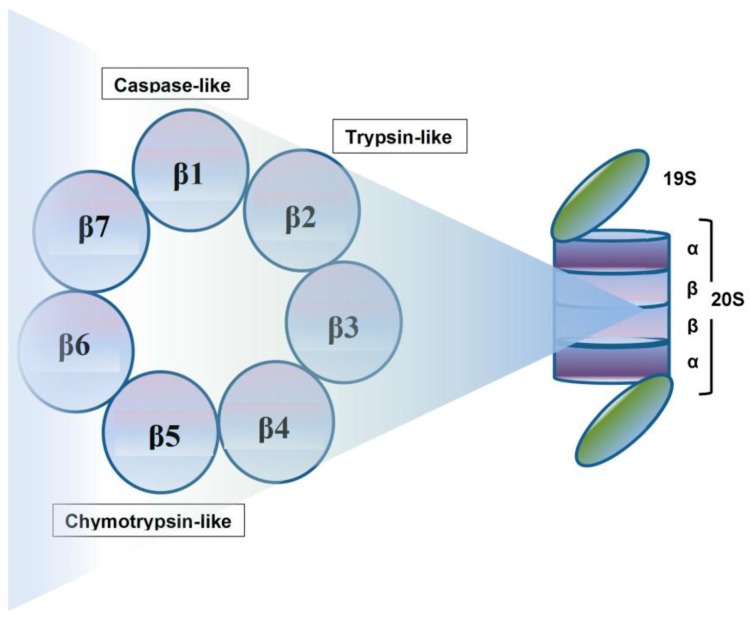
The structure of the 26S proteasome comprises a 20S core that contains active enzymatic sites with chymotrypsin-like (β5), trypsin-like (β2) and caspase-like (β1) activities, and a 19S cap at either end [11].

Crucial to several processes regulating cellular function and homeostasis is the ubiquitin-proteasome system (UPS) [22,23]. The proteasome, an integral component of UPS [23,24,25], is responsible for regulation and degradation of the majority of intracellular proteins. Proteins targeted for proteasomal degradation are polyubiquitinated by a set of enzymes, including the ubiquitin-activating enzyme E1, ubiquitin-conjugating enzyme E2, and the ubiquitin E3 ligases [22,26,27,28]. The protein carrying a polyubiquitinated chain is recognized by the 19S subunit and degraded into small peptides by the 20S proteasome [29], and this multi-subunit protease complex is referred to as 26S proteasome. The 19S regulatory units flank or “cap” the barrel-shaped 20S core particle, consisting of four highly homologous rings that enclose a central catalytic chamber with proteolytic active sites (Figure 1). Each of the rings contains seven subunits α and β, which are arranged one above the other in the order of α-β-β-α [30]. While the outer two α-rings surround a small opening through which only denatured polypeptide substrates may pass, two central β-rings contain multiple proteolytic sites that function together in protein degradation [31,32]. Each of these two β rings comprises three proteolytic sites-β1 (caspase-like, C-L), β2 (trypsin-like, T-L) and β5 (chymotrypsin-like, CT-L) [33,34]. The 26S proteasome is highly abundant and ubiquitous in cells; for example, Lightcap et al. reported there were 2.7 × 10^−19^ moles of 20S proteasome in a red blood cell and 8 × 10^−19^ moles in a white blood cell, with equivalent proteasome concentrations of 1.9–4.1 and 1–6 mmol/L, respectively [35].

**Figure 2:**
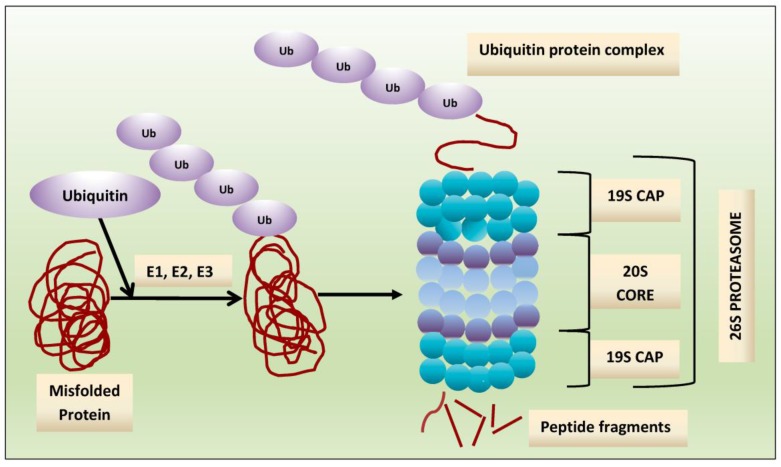
Ubiquitin-Proteasome System. A chain of ubiquitin moieties is attached by the action of a series of ubiquitin ligases (E1, E2, E3) to lysine residues on the target protein to be degraded. The ubiquitin-protein complex is transported to the proteasome, where the ubiquitin chain is removed, allowing the target protein to be unfolded and translocated to the interior of the proteasome, where it is degraded by 3 threonine proteases to yield peptide fragments. Adapted from Molineaux [67].

Exposing cells to few stimuli, such as interferon-γ, tumor necrosis factor-α (TNF-α) and bacterial lipopolysaccharides, induces the synthesis of other catalytic subunits that are incorporated together into an alternative proteasome form known as the immunoproteasome, which is preferentially expressed in cells of lymphoid origin and generates antigenic peptides presented by class I major histocompatibility complex to induce a cytotoxic immune response [50,51,52,53]. In the 20S immunoproteasome (i20S), proteolytically active subunits β1, β2 and β5 are substituted by their equivalents β1i (LMP2), β2i (MECL-1) and β5i (LMP7) [54]. Although proteasome contains multiple catalytic sites, to inhibit its function at the constitutive or immunoproteasome level, it is sufficient to block only the β5/LMP7 subunit (CT-L) [55,56]. Compared to the constitutive 20S proteasome, the immunoproteasome 20Si possesses enhanced chymotrypsin- and trypsin-like activities and reduced caspase-like activity. Proteins intended for degradation including incorrectly folded proteins, and proteins with short half-life and mostly regulatory function are cut into oligopeptide chains with an average length of 8–12 amino acids [57,58] (Figure 2).

**Figure 3:**
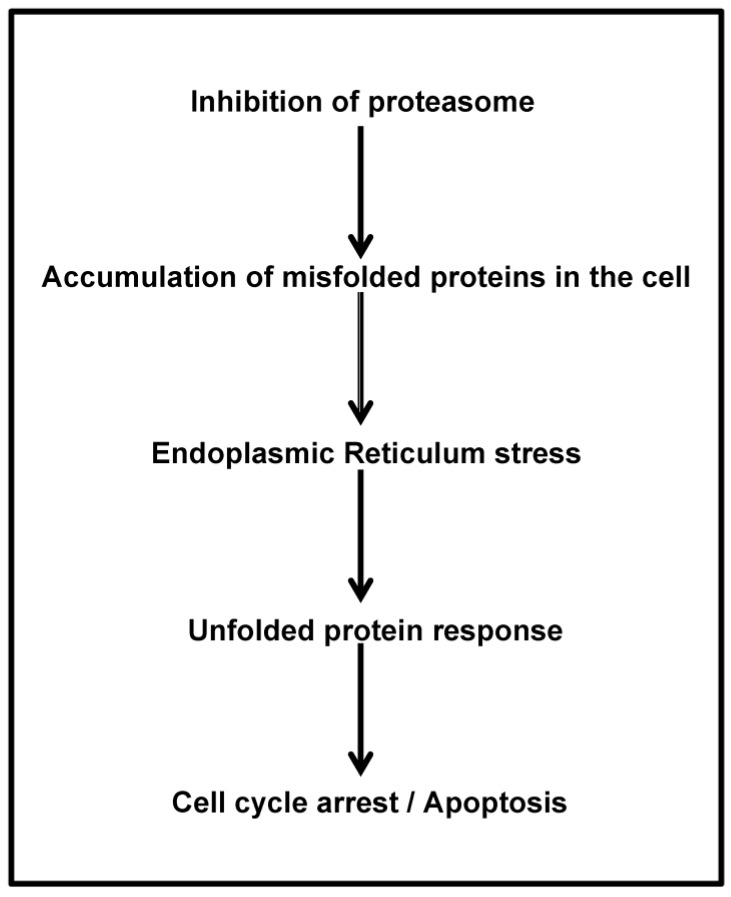
Consequences of proteasome inhibition. Adapted from Franken [68].

As both normal and malignant plasma cells (PCs) are highly secretory cells, they require a well-developed endoplasmic reticulum (ER), expansion of secretory apparatus and production of chaperone proteins that ensure proper immunoglobulin (Ig) translation and folding [59]. A stress signaling pathway called the unfolded protein response (UPR) ensures that PCs can handle the proper folding of proteins and prevent the aggregation of misfolded proteins. These proteins are then transported out of the ER and degraded by proteasome and this process is referred to as ER-associated degradation (ERAD) [60,61]. Treatment of MM cells with PIs results in the accumulation of misfolded Ig within the ER, because of inhibition of proteasomal function [62]. Such stress activates the UPR pathway, which is mediated by activation or translational repression of several transcription factors, such as XBP-1, ATF6 and PERK/eIF2a [62,63]. Generally, UPR allows the cell to survive temporary but reversible environmental conditions, such as chemical insult or nutrition deprivation. However, during prolonged stress such as caused by PIs, UPR activation leads to cell cycle arrest [64] and induction of apoptosis [65] (Figure 3). PIs initiate UPR leading to apoptosis preferentially in cells with high Ig production and thus, partial inhibition of proteasome in vivo is not toxic to normal cells, but is sufficient to kill the myeloma cells [66].

## 3. Proteasome Inhibitors: Mechanisms of Anti-Myeloma Effect

The success of PIs in MM is dependent on their pleiotropic effects, which decrease growth and survival of MM cells and the interaction between MM cells and bone marrow (BM) microenvironment (cellular adhesion, angiogenesis and cytokine circuits). Several shared mechanisms of action of PIs have been identified based on the preclinical studies.

**Figure 4:**
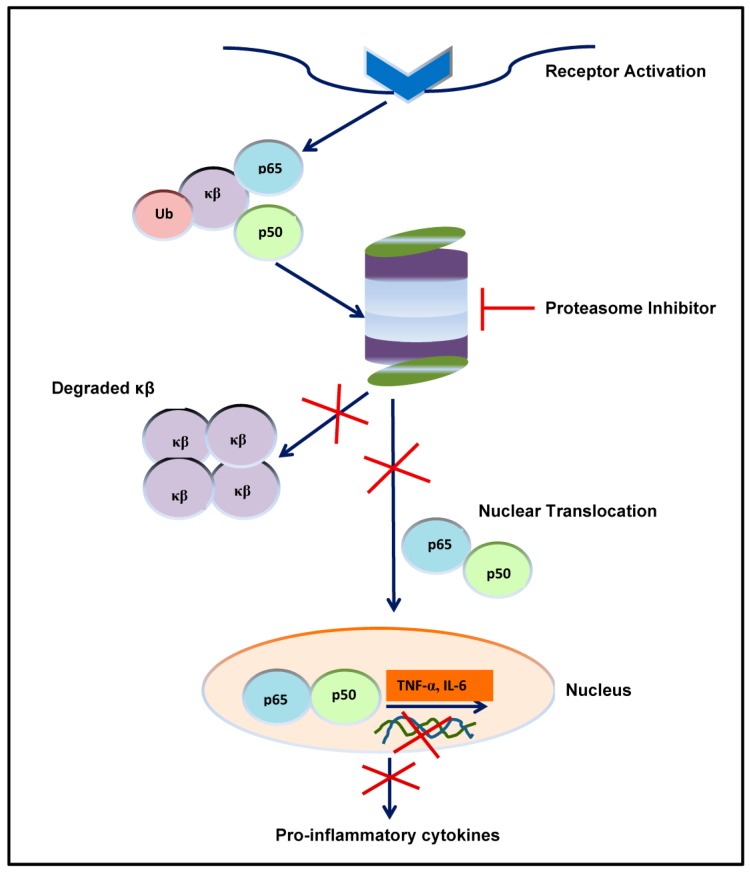
Blockade of nuclear factor (NF)-κB activation by the proteasome inhibition. Inhibition of proteasome prevents the degradation of the natural inhibitor of NF-κB (i.e., IκB) along with nuclear translocation of p50/p65 and transcription of pro-inflammatory cytokines. Adapted from Verbrugge, [69]. TNF-α: tumor necrosis factor-α.

Proteasome inhibition prevents the clearance of misfolded proteins, inducing “ER stress” or impaired ERAD and activation of the UPR [57,70], which in turn leads to apoptosis and cell death [71]. Proteasome is responsible for the degradation of several pro-apoptotic and tumor suppressor proteins that become upregulated by its inhibition and lead to apoptosis: deregulation of the turnover of cyclins and disruption of cyclin-dependent kinase activity; Jun *N*-terminal kinase (JNK) stabilization and Fas upregulation; stabilization of p53; and a shifting of the pro-apoptotic and anti-apoptotic balance in the Bcl-2 family of proteins and inhibition of nuclear factor-κB (NF-κB) pathway [5]. NF-κB is a transcription factor that plays an important role in tumorigenesis by suppressing apoptosis, inducing angiogenesis and proliferation, and by enhancement of tumor cell invasiveness and metastasis [72]. MM cells have elevated levels of NF-κB [73,74] and proteasome inhibition leads to apoptosis of tumor cells by blocking NF-κB nuclear translocation and gene transcription [73,74,75,76,77]. PIs block the NF-κB pathway by preventing degradation of IκB (NF-κB inhibitor) after its polyubyquitination by IKK (IκB kinase), [78] thereby preventing NF-κB to enter the nucleus and activate downstream pathways (Figure 4). Tumor suppressor molecule p27 is able to arrest progression through the G1/S phase of cell cycle and the accumulation of this protein due to proteasome inhibition results in cell cycle arrest and apoptosis [79]. Moreover, PIs induce an upregulation of the pro-apoptotic factors p53, Bax and NOXA, whereas anti-apoptotic proteins such as Bcl-2 and inhibitor of apoptosis proteins (IAP) are reduced [80].

## 4. Clinical Development of Novel Proteasome Inhibitors

### 4.1. Carfilzomib

Carfilzomib (CFZ) is an irreversible peptide epoxyketone class PI that is structurally and mechanistically distinct from BTZ. It is the most specific and potent PI. CFZ selectively inhibits the chymotrypsin-like activity of the proteasome [81] through the inhibition of its β5 subunit. The irreversible binding by CFZ leads to more sustained activity compared to BTZ, as new subunit synthesis and proteasome assembly are required for restoration of proteasome activity, what occurs in approximately 24 h for nucleated cells [81]. CFZ has a great specificity against the β5 subunit, but the affinity for other β-subunits (β1, β2) is lower, so the trypsin-like and caspase-like activities of proteasome are less inhibited. In addition to targeting β5 subunit in the constitutive 20S proteasome, CFZ also targets β5i subunit of the immunoproteasome 20Si (LMP7 subunit) [56,82]. CFZ induces apoptosis of BTZ-naive and even BTZ-pre-treated MM cells and is also more effective in xenograft models, which is consistent with its higher affinity for the proteasome [81,82]. CFZ also showed minimal off-target activity against other enzymes such as serine proteases including cathepsin G, cathepsin A, chymase, dipeptidyl peptidase II and Htra2/Omi, which may be responsible for its differential toxicity profile compared with BTZ [18,56]. These biological differences confer an improved activity shown in preclinical work and confirmed by clinical trials demonstrating CFZ overcoming BTZ-resistance [82]. CFZ has been shown to be active against BTZ-resistant MM cell lines, as well as samples from MM patients with clinical resistance to BTZ [3]. CFZ can also overcome resistance to other conventional agents and acts synergistically with dexamethasone (DEX) to enhance cell death [82]. Preclinical and early clinical work with CFZ confirmed rapid clearance of CFZ from plasma with a half-life (t_1/2_) of <30 min with widespread tissue distribution with the exception of the brain [81,83]. Proteasome activity in blood and peripleral blood mononuclear cells (PBMCs) was inhibited by CFZ in a dose-dependent manner; >75% inhibition at 15 mg/m^2^ after the first dose and >90% inhibition after five doses. As predicted by the binding properties of CFZ, proteasomal inhibition persisted after the drug had been cleared. Proteasome function in PBMC eventually recovered more than 2 weeks after dosing. The administration schedule of CFZ is based on the in vivo study by Demo et al. [81] that showed that CFZ was well tolerated when administered for either 2 or 5 consecutive days and the anti-tumor efficacy of CFZ delivered on 2 consecutive days was greater than that of BTZ administered on its usual clinical dosing schedule with 2 days rest after each dose. This may be due to the greater efficacy of a sustained inhibition of the proteasome for 48 h, and led to the choice of the two consecutive days’ weekly schedule in clinical trials of CFZ.

The US Food and Drug Administration (FDA) granted accelerated approval to CFZ in 2012 for the treatment of relapsed or refractory MM (RRMM) patients who have received at least two prior therapies, including BTZ and an IMiD, and have demonstrated disease progression on or within 60 days of the completion of the last therapy [2]. This was based on efficacy results from the single-arm trial PX-171-003-A17 and combined safety data from four phase II studies (PX-171-003-A0, PX-171-003-A1, PX-171-004, and PX-171-005) [84]. PX-171-003-A0 included 46 MM patients relapsing after ≥2 prior lines of therapy, all being refractory to the last line of therapy [85,86]. CFZ 20 mg/m^2^ was administered on days 1, 2, 8, 9, 15 and 16 of 28 days’ cycles for up to 12 cycles. Overall response rate (ORR) was 17% and clinical benefit rate (CBR) was 24% (5 patients achieved partial response (PR) and 5 minimal response (MR)). These results led to an expansion phase (PX-171-003-A1) that included 266 patients who had previously received BTZ, an IMiD and an alkylator [36,85]. Ninety five percent patients were refractory to their last treatment and 80% were intolerant of or refractory to both BTZ and lenalidomide (LEN). The dose and schedule of treatment were identical except that the dose was increased to 27 mg/m^2^ from cycle 2. ORR was 24% with a median duration of response (DOR) of 7.8 months, median progression-free survival (mPFS) was 3.7 months and median overall survival (mOS) was of 15.6 months, compared with the 9-month OS in the previously mentioned BTZ and LEN-refractory cohort studied by Kumar et al. [87]. Responses were also seen in BTZ-refractory MM patients. CFZ was well tolerated without cumulative toxicity: most grade 3–4 (G3–4) adverse events (AEs) were hematologic and only 12% of patients experienced neuropathy. A phase II (PX-171-004) trial evaluated CFZ in BTZ-naive patients with RRMM with a median of two prior therapies [88]. A total of 129 patients received CFZ on days 1, 2, 8, 9, 15 and 16 in a 28-days cycle. The first cohort of 59 patients received a dose of 20 mg/m^2^. A second cohort of 70 patients escalated to 27 mg/m^2^ in cycle 2 after an amendment due to the results of the PX-171-002 study. ORRs were higher than in the previous studies with BTZ-refractory patients and were 42% and 52% in cohorts 1 and 2, respectively. Median DOR was 13.1 months for cohort 1 and not reached (NR) for cohort 2 and PFS was 8.3 months and not reached, respectively. All of these results were also higher than in BTZ-refractory patients. AEs were comparable with previously reported rates at the same dose levels. The study showed that single agent CFZ is even more effective in patients who are BTZ-naïve than patients with RRMM who have received BTZ, with similar toxicity [88]. CFZ has been evaluated in MM patients with renal insufficiency in the PX-171-005 trial [85]. CFZ was dosed at 15 mg/m^2^ IV on days 1, 2, 8, 9, 15, 16 (28 days cycle), 20 mg/m^2^ IV in cycle 2 and 27 mg/m^2^ IV in cycle 3. Patients with a suboptimal response, i.e., failing to achieve PR by cycle 2 or complete response (CR) by cycle 4 could receive DEX in addition. Thirty nine patients with different degrees of renal impairment were enrolled. Pharmacokinetic (PK)/pharmacodynamic (PD) parameters were similar across all groups. CFZ was undetectable in plasma within 3 h and did not accumulate after cycle 2. A quarter of the patients had ≥PR (*n* = 8), while five had MR and 13 remained with stable disease (SD).

The question of dose-response relationship of CFZ was explored in a phase I trial in 55 relapsed MM patients in combination with (*n* = 22) or without DEX 40 mg weekly (*n* = 33). Papadopoulos et al. confirmed the safety of a dose-escalation regimen with CFZ dose range between 36 and 70 mg/m^2^ and infusion time of 30 min (infusion was over 2–10 min in other CFZ studies) [89]. The maximum tolerated dose (MTD) was determined as 56 mg/m^2^. Furthermore, although the cohort was much smaller, ORR was 50% compared with 24% in PX-171-003-A1 trial. Toxicity was higher than reported for 27 mg/m^2^: nausea, dyspnea and fatigue (but not PN) were the most common AEs but were largely G1–2. Most G3–4 events were hematologic. In combination with DEX (40 mg/weekly), the toxicity profile was more favorable than single-agent administration at the same doses (less nausea, vomiting, diarrhea, pyrexia, dyspnea, fatigue, and creatinine elevation), but some AEs were more frequent with the combination (headache, hypertension, and upper respiratory tract infection). Higher doses were well tolerated if infusion time was prolonged to 30 min, with increased efficacy [68]. Lendvai et al. conducted a phase II study of CFZ 56 mg/m^2^ with the option of adding DEX in 44 RRMM patients [90]. Patients were pretreated with a median of five prior regimes, including at least one regimen with BTZ. Of the 42 evaluable patients, 55% achieved at least PR. PFS, DOR and OS were 4.1, 11.7 and 20.3 months, respectively. Of the 6 patients who responded but later progressed and had DEX added four had SD. CFZ 56 mg/m^2^ ± DEX was tolerable: seven patients discontinued treatment due to AEs which included left ventricular systolic dysfunction (*n* = 5), fever (*n* = 1), and myelodysplastic syndrome (*n* = 1).

The escalated dosing schedule of CFZ was further investigated in patients with previously treated progressive MM, not refractory to prior BTZ therapy, in the ENDEAVOR trial. This trial randomized 929 patients with RRMM and was the first of the two phase III studies comparing the efficacy of two PIs: CFZ versus BTZ [37]. The median number of prior therapies was two, with 54% of patients pretreated with BTZ in both groups. CFZ was given at 20/56 mg/m^2^ IV days 1, 2, 8, 9, 15, 16 over 30 min infusion and BTZ at 1.3 mg/m^2^ IV/SC days 1, 4, 8, and 11 and DEX 20 mg weekly. Patients who did not achieve PR on prior PI or had <6 months PI-free interval were excluded (54% had prior PI exposure). ORR was 77% in the CFZ group and 63% in the BTZ group (*p* < 0.0001), including at least very good partial response (VGPR) in 54% and 29% of patients, respectively. With a median follow-up of 12 months, a 9-month PFS advantage was seen in the CFZ group (18.7 months vs. 9.4 months, HR 0.53; *p* < 0.0001). Furthermore, DOR was also doubled (21.3 vs. 10.4 months). OS difference was not seen due to the relatively short follow up, although a trend favoring CFZ was noted. A PFS benefit was seen in both BTZ-exposed and BTZ-naive patients in the subset analysis. Treatment discontinuation due to AEs occurred with similar frequency in the two groups (14% vs. 16%). The rate of polyneuropathy in the CFZ group was significantly lower (9% versus 27% in BTZ group; odds ratio 0.14; *p* < 0.0001). The study was criticized for evaluating patients who had previously received BTZ (nearly half the study population) and comparing those being re-treated with BTZ with those given a more potent second-generation CFZ. The group receiving CFZ expectedly achieved better outcomes as the study answered the question whether, in relapsed patients, it was better to re-treat with BTZ in the previously exposed or to use CFZ instead. The question that needs to be answered is if the patients who received BTZ before could receive CFZ at relapse and obtain the same survival benefit as patients who start out with CFZ in the frontline therapy.

Single-agent CFZ has an acceptable safety profile in heavily pretreated patients with RRMM. For a broad look at safety, a pooled analysis of 526 patients treated with single-agent CFZ in 3 phase II studies (PX-171-003, PX-171-004 and PX-171-005) revealed fatigue (55%), anemia (47%), nausea (45%), thrombocytopenia (36%), dyspnea (35%), diarrhea (33%) and pyrexia (30%), cardiac events (22%), respiratory events 69% (dyspnea 42%), but PN in only 14% (generally G1–2) [91]. The G3 AEs present in ≥10% of patients were mostly hematologic: thrombocytopenia (23%), anemia (22%), lymphopenia (18%), pneumonia (11%) and neutropenia (10%). Of note, only 14% developed PN (G3, 1.3%). Dose modifications or discontinuations were required in only five patients. Essentially no patients required discontinuation or dose adjustments due to neurotoxicity, permitting long-term treatment with CFZ. Renal AEs (mainly G2) were reported in 174 (33%) patients, but CFZ was discontinued because of a renal AE in only 21 patients (4%), which is in line with the PX-171-005 study [92]. Cardiac events were reported in 7% of patients, regardless of causality. Cardiac events resulting in treatment discontinuation included congestive heart failure (2%), cardiac arrest (1%), and myocardial ischemia (<1%). The extent to which cardiac events were due to patients’ baseline comorbidities, toxicity from prior treatments, effects of MM, CFZ itself, or a combination of these factors could not be determined. The rates and causes of death were consistent with those observed in heavily pretreated patients with end-stage MM. 

Clinical trials with the combination of CFZ with an IMiD, LEN and DEX for treatment of relapsed MM patients have been conducted. A phase III ASPIRE trial evaluated the combination of CFZ with LEN-DEX (KRd) in comparison with LEN-DEX (Rd) [38]. A total of 792 RRMM patients were randomized between KRd and Rd. CFZ was administered IV on days 1, 2, 8, 9, 15, and 16 (20/27 mg/m^2^) cycles 1–12 and days 1, 2, 15, and 16 of cycles 13–18, after which only Rd was continued. Patients who were previously progressed on BTZ or were LEN-refractory were excluded. The median number of prior lines of treatment was 2 in both groups. Of all patients, 66% had previously been treated with BTZ and 20% with LEN. The CFZ group demonstrated improved ORR (87%; CR in 32%) compared to the control group (67%; CR in 9%; *p*<0.001). This translated into significantly improved PFS in the CFZ group (median, 26.3 months) compared with the control group (17.6 months, *p* = 0.0001). The PFS benefit was seen in most subgroups analyzed, including in patients with previous refractoriness to IMiD, but not in those with previous BTZ resistance. The trial also demonstrated an OS advantage at 2 years for the CFZ group over the control group (73% vs. 65%; *p* = 0.04), but it is important to acknowledge that only 2% of the patients had access to CFZ upon progression [93] and therefore, survival interpretation should be made with caution. ORR in the KRd group was higher than the 69% reached in the previous phase II study. However, the latter study included more heavily pretreated patients, including more BTZ-(25%) and LEN-(44%) refractory patients. Furthermore only 27% of patients in the phase II trial were LEN-naïve compared with over 80% in this trial. AEs of G≥3 were reported in 84% in the KRd group and 81% in the control group. Grade 3–4 AEs of interest that were more frequent in the KRd group were cardiac failure (3.8% vs. 1.8%), ischemic heart disease (3.3% vs. 2.1%), and hypertension (4.3% vs. 1.8%). A number of common AEs were reported at a higher rate in the CFZ group than in the control group, including diarrhea, cough, fever, and hypertension. Serious AEs (SAEs), including cardiac events, were reported more frequently during the first 18 cycles of treatment than in later cycles. The rate of renal failure was comparable between the two treatment groups. Only 3.3% of patients developed G ≥ 3 renal failure in the KRd group, which is dramatically lower than 17% reported in the FOCUS trial (see below). This trial led to FDA approval of CFZ in combination with LEN-DEX for treatment of patients with RRMM who have received 1–3 prior lines of therapy [94]. Given the success with KRd, a phase I study looked at the combination of CFZ with a newer IMiD, pomalidomide (POM) and low-dose DEX [95]. Patients were planned for six cycles of therapy after which maintenance with CFZ and POM was allowed. A total of 32 RRMM patients, most of whom were dual-refractory, were enrolled. Patients had received a median of six prior lines of therapy. DLT was seen at CFZ 27 mg/m^2^, POM at 4 mg/d and DEX 40 mg weekly. Patients were planned for six cycles of therapy after which maintenance with CFZ and POM was allowed. ORR was 50% (VGPR in 16%). The mPFS and mOS were 7.2 and 20.6 months, respectively. AEs were mostly hematologic and were comparable with KRd. 

In the phase III FOCUS trial 315 patients with RRMM were randomized between single-agent CFZ and low-dose steroids (prednisone (PRED) or DEX) with or without continuous low-dose oral cyclophosphamide (Cy) [40]. All patients were previously treated with BTZ, LEN, steroids and alkylating agent. A total of 62% and 63% patients were BTZ- and LEN-refractory in the CFZ and control group, respectively. CFZ was administered in a 28-days cycle at 20/27 mg/m^2^. The great majority of patients (95%) received Cy. The primary endpoint of OS was not met: in the CFZ group, it was 10.2 months compared with 10 months (*p* = 0.42) in the control group. ORR was slightly improved with CFZ (19% vs. 11%; *p* = 0.03), although it did not translate into PFS benefit (3.7 vs. 3.3 months, *p* = 0.25, for the CFZ and control groups, respectively). AEs were similar to those reported in previous early phase studies with the exception of higher rates of renal failure: 17% of G ≥ 3 renal failure with CFZ versus 5% in the control group. The incidence of heart failure (4.5%) was similar to that previously reported studies, but the difference in the control group (0.7%) was striking, implicating cardiotoxicity of CFZ [68]. The failure of the study to meet its primary endpoint was probably due to the efficacy of Cy in the control group [68].

With the objective of improving the convenience of administration, a multicenter phase I/II CHAMPION-1 study explored once-weekly schedule of CFZ in RRMM patients [39]. Patients (*n* = 116) received CFZ (30 min IV infusion) on days 1, 8 and 15 in 28-days cycle (20 mg/m^2^ on day 1 of cycle 1, followed by escalating doses of 45, 56, 70 and 88 mg/m^2^) and DEX 40 mg weekly. MTD was established at 70 mg/m^2^. A total of 104 patients received CFZ 70 mg/m^2^; the median number of prior therapies was 1 and 55% patients were BTZ-refractory. At 70 mg/m^2^, ORR of 77% and mPFS was 12.6 months. AEs were comparable to the twice weekly schedule and the most common G ≥ 3 AEs were fatigue (11%) and hypertension (7%). The results of the CHAMPION study have led to the phase III ARROW trial which is currently enrolling RRMM patients who have had 2–3 prior therapies (including both BTZ and IMiD) with the objective of comparing the 70 mg/m^2^ weekly dosing of CFZ with the approved two consecutive days’ dosing (20/27 mg/m^2^) schedule in combination with weekly DEX (in both arms) [68]. This trial will enroll 460 RRMM patients and the primary end-point is ORR.

With the activity of CFZ in RRMM being evident with manageable toxicity profile, clinical trials of CFZ have been conducted for frontline treatment of treatment-naïve newly diagnosed MM (NDMM) patients. In a phase II trial, the combination of CFZ (20/36 mg/m^2^, standard dosing days), Cy 300 mg/m^2^ days 1, 8, 15, and DEX 40 mg weekly was evaluated in 58 transplant-ineligible NDMM patients [43]. Following nine induction cycles, CFZ maintenance at 36 mg/m^2^ was given on two consecutive days every 2 weeks until progression or intolerance. Seventy four percent patients proceeded to maintenance, which was given for a median of 9 months. ORR was 95%, with 71% of patients achieving ≥VGPR. Depth of response increased with the duration of treatment. The 24-month PFS and OS were 76% and 87%, respectively. In a phase Ib/II dose-escalation/expansion single-arm CYKLONE trial for treatment of NDMM, CFZ was evaluated in combination with Cy (300 mg/m^2^, days 1, 8, 15), thalidomide (THAL; 100 mg/d, days 1–28), and DEX (40 mg weekly) in 64 NDMM patients [42]. MTD of CFZ was determined at 20/36 mg/m^2^ (20 mg/m^2^ in cycle 1; 36 mg/m^2^ thereafter). The median number of cycles administered was 4. ORR across all dose levels was 91%, including ≥VGPR in 59% of patients. Time to response was rapid, with 81% and 23% of patients achieving PR and VGPR, respectively, by the end of cycle 1. Stem cell mobilization was successful, but autologous hematopoietic cell transplantation (AHCT) was performed in 53% patients, who were all transplant-eligible. Two-year PFS and OS were 76% and 96%, respectively.

Two trials have assessed KRd regimen for NDMM. A phase I/II study conducted by Jakubowiak et al. that tested this three-drug combination to determine the MTD. Patients (*n* = 53) received three dose levels of CFZ (20, 27, or 36 mg/m^2^), combined with LEN (25 mg days 1–21) and DEX (40/20 mg weekly) [44]. Transplant-eligible patients were collected after four cycles and were allowed to proceed to early AHCT. The median number of cycles was 12 (range, 1–25 cycles). ORR was 100% (≥VGPR in 91%). Responses deepened with continued treatment and were similar across the three dose levels. In 36 patients completing at least 8 cycles, 78% achieved at least near CR (nCR) and 61% stringent CR (sCR). The 3-year PFS was 80% and OS 100%. Successful stem cell collection was achieved in all the 18 patients contemplating AHCT. Seven of 35 transplant-eligible patients proceeded to AHCT. Regarding the toxicity profile, low rates of neutropenia (12% of G3–4) were observed, and 24% of patients had PN that was limited to G1/2 in all cases. In another study, triple regimen of CFZ (20/36 mg/m^2^), LEN (25 mg days 1-21) and DEX (20/10 mg on days of CFZ) was evaluated in 45 NDMM patients and in 12 high-risk smoldering MM patients [96]. Depth of response improved with duration of treatment and reached a plateau after 8 cycles. ORR in NDMM was 98% with at least VGPR achieved in 90%. Minimal residual disease (MRD) status assessed by multi-parameter flow cytometry (MPFC) and/or next generation sequencing in patients achieving at least nCR showed a high rate of MRD negativity, which correlated with superior PFS. Another combination that has been evaluated in a phase II European Myeloma Network study is CFZ plus THAL-DEX (KTd) as induction/consolidation therapy for transplant-eligible NDMM patients (*n* = 91) [97]. Patients received 4 induction cycles of KTd; CFZ 20/27 mg/m^2^ (*n* = 50), 20/36 mg/m^2^ (*n* = 20), 20/45 mg/m^2^ (*n* = 21), or 20/56 mg/m^2^ (*n* = 20) on days 1, 2, 8, 9, 15, and 16 of 28-days cycle, THAL 200 mg on days 1-28 and DEX 20 mg on days 1, 2, 8, 9, 15, and 16. After AHCT, patients had KTd consolidation. Common G3/4 AEs included respiratory (15%), gastrointestinal (GI; 12%), and skin disorders (10%), whereas PN was rare (1%). ORR after induction and consolidation were 90% and 96%, respectively, and the corresponding CR rates were 25% and 63%. At a median follow-up of 23 months, 3-year PFS was 72%.

An important question is whether CFZ can displace BTZ from the front-line therapy of MM. To answer this relevant question, a landmark phase III CLARION study was conducted that randomized 955 transplant-ineligible NDMM patients to receive CFZ, melphalan (MEL) and PRED (KMP) or BTZ, MEL and PRED (VMP) for 54 weeks. The median patient age was 72. MEL 9 mg/m^2^ and PRED 60 mg/m^2^ were administered orally on days 1–4 of each 6-weekly cycle. CFZ 20/36 mg/m^2^ (20 mg/ m^2^ on days 1 and 2 of cycle 1) was given IV on days 1, 2, 8, 9, 22, 23, 29, 30, and BTZ 1.3 mg/m^2^ was administered IV or subcutaneously (SQ) on days 1, 4, 8, 11, 22, 25, 29, 32 of cycles 1–4 and days 1, 8, 22, 29 of cycles 5–9. Survival analysis demonstrated a lack of superiority of CFZ over BTZ. Median PFS was 22.3 months in CFZ group versus 22.1 months in BTZ group. In addition, the rate of fatal treatment emergent-AEs was higher with CFZ versus BTZ (6.5% vs. 4.3%, respectively). Grade ≥3 AEs were experienced by 75% and 76% patients, respectively, though G≥2 PN was reported in 2.5% of CFZ-treated patients compared with 35% with BTZ. Another randomized phase III ENDURANCE (E1A11) study, being conducted by ECOG-ACRIN, is comparing CFZ with BTZ in combination with LEN-DEX (KRd vs. VRd) for NDMM patients, which reflects the current practice patterns in the United States where VRd is the widely established standard-of-care for NDMM. The study is currently recruiting subjects and has OS as the primary endpoint. 

### 4.2. Ixazomib

Ixazomib (MLN9708) is a dipeptidilic boronic acid PI that is rapidly hydrolyzed in water and converts into MLN2238, the active moiety that selectively and reversibly inhibits proteasome. It is the first orally bioavailable PI to be used in the clinic. MLN9708, like BTZ, specifically inhibits the proteolytic β5 subunit of the 20S proteasome [98]. At higher concentrations, MLN9708 also inhibits the proteolytic β1 and β2 subunits and causes accumulation of ubiquitinated proteins [98]. MLN9708 also targets immunoproteasome [99]. Preclinical studies have shown that MLN9708/MLN2238 has a similar selectivity and potency in terms of proteasome inhibition to BTZ, but proteasome-binding kinetics are different. Both MLN9708 and BTZ show time-dependent reversible proteasome inhibition, although proteasome dissociation half-life (t1/2) for MLN9708 is six-fold faster than that of BTZ (18 vs. 110 min) [98]. In vitro studies performed with different human MM cell lines treated with various concentrations of MLN9708 showed a concentration-dependent decrease in the viability of all treated cell lines [100]. Furthermore, MLN9708 induces apoptosis of MM cell lines resistant to conventional therapies and in primary MM cells obtained from BTZ- and LEN-resistant patients [101]. MLN9708 leads to an increase of ubiquitinated proteins in MM cell lines (MM.1S); an increase in cleavage of poly(ADP)ribose polymerase (PARP), a marker of apoptosis; an activation of caspase-3, -8 and -9; upregulation of signaling pathways such as p53-p21, p53-NOXA-PUMA and Rb-E2F; activation of ER stress response proteins and inhibition of NF-κB pathway [101]. NF-κB inhibition reduces the release of cytokines by bone marrow stromal cells important for growth and survival of MM cells, thereby disrupting the cytoprotective effects of BM microenvironment on MM cells [101]. Furthermore, MLN9708 inhibits tumor-associated angiogenic activity as it reduces the expression of the angiogenesis markers VEGFR2 and PECAM [101]. MLN9708 also induces the expression of binding immunoglobulin protein and CCAAT-enhancer-binding protein homologous protein transcription factor—both connected to ER stress response. MLN9708 also induces expression of Bip and CHOP-heat shock protein and transcription factor connected with ER, the expression of which is induced by cellular stress and is involved in mediating apoptosis. In vitro studies showed synergistic anti-myeloma activity of MLN9708 with DEX, LEN and HDACi such as vorinostat [98]. In murine MM xenograft models, MLN9708 induced significant inhibition of tumor growth and prolonged survival compared with control mice [101]. In vivo studies in two xenograft models showed that both MLN9708 and BTZ had a similar maximum level of proteasome inhibition (Emax) in blood samples [98]. BTZ showed a greater blood area under the effect-time curve (AUE), while MLN9708 showed higher and more sustained proteasome inhibition in tumor tissue of both xenografts models with a higher tumor/blood AUE ratio [102]. At the respective MTD, MLN9708 showed a larger blood volume distribution at steady state compared with BTZ, indicating MLN9708 moves more rapidly from blood to the tissue compartment leading to greater PD effects in tissues [98]. 

MicroRNA profiling in MLN9708-treated MM cells shows increased expression of miR33b, which has constitutively low expression in MM cells. The overexpression of this gene plays a critical role in the tumor growth inhibition and prolonged survival of mouse xenograft models [103]. Increased miR33b expression leads to cell death and apoptosis by blocking proto-oncogene (PIM-1) via inhibiting serine/threonine kinase activity. Increased PIM-1 expression leads to significant abrogation of miR33b-induced cell death. Upregulation of miRNA is associated with reduced migration and viability of MM cells as well as with increased apoptosis and sensitivity of MM to MLN9708 and, in addition, in human MM xenograft models, miR33b inhibits tumor growth and prolongs survival. All these suggest that miR33b is a tumor suppressor that plays a role in MLN9708-induced apoptotic signaling in MM cells [103].

Data from phase I studies of MLN9708, administered IV and PO, using twice weekly and weekly schedules, demonstrated approximately dose-linear PK over the higher range of doses tested (1–2.34 mg/m^2^) [104]. Oral administration resulted in rapid absorption (T_max_ ~1 h) and systemic exposure similar to those achieved with IV dosing on the same schedule indicating a substantial bioavailability [105]. PK results also indicate that MLN9708 administration at a flat dose is feasible [85] and that makes oral administration possible [106]. Two phase I studies evaluated MLN9708 given IV in solid tumors and lymphoma: C16001, a dose-escalation study of a biweekly administration in 67 patients with non-hematologic malignancies that set the MTD for the biweekly schedule at 1.76 mg/m^2^ and C16002, a study of weekly MLN9708 in advanced lymphoma patients who had received ≥2 prior lines of therapies: 30 patients with a range of histologies received MLN9708 0.125–3.11 mg/m^2^ on days 1, 8 and 15 of 28 days cycles [85]. MTD was determined to be 2.34 mg/m^2^. Most common drug-related AEs included fatigue (43%), diarrhea (33%), nausea, vomiting and thrombocytopenia (27% each). G3–4 AEs included neutropenia (20%), thrombocytopenia (13%) and diarrhea (10%). MLN9708-related PN occurred in four (13%) patients; no G3–4 events were reported. Plasma exposure increased dose proportionally from 0.5 to 3.11 mg/m^2^; terminal half-life was 4–12 days after multiple dosing. Of 26 evaluable patients, five achieved responses: one CR and four PR. Data suggested weekly IV MLN9708 was well-tolerated overall [107]. 

As monotherapy in RRMM patients, oral MLN9708 was evaluated in two phase I studies [108,109] using either weekly [108] or biweekly dosing schedule [109]. Furthermore, patients enrolled in the dose-escalation phase of both trials had received ≥2 prior lines of therapy, including BTZ and IMiD [108,109]. Kumar et al. [108] enrolled 60 patients (2009–2012) in a study (C16004) primarily aimed at determining the MTD, safety and tolerability of MLN9708 and, secondarily, ORR and PK. Thirty two patients received oral MLN9708 on days 1, 8 and 15 of a 28-days cycle (up to 12 cycles) at doses ranging from 0.24 to 3.95 mg/m^2^. An MTD of 2.97 mg/m^2^ was used in 31 patients of four expansion cohorts based on relapsed/refractory status and prior BTZ and CFZ exposure. The median number of prior regimens was four (range, 1–13); 85% patients had received BTZ, 77% had prior AHCT, 72% were refractory to the last therapy. The most common G3–4 hematologic AEs were thrombocytopenia (33%) and neutropenia (18%). Non-hematologic AEs mainly included diarrhea (17%), nausea and vomiting (~5%). Only one patient developed G3 PN and 12% discontinued therapy due to AE. Anti-MM activity was encouraging: 18% had at least PR, while DOR was 7.3 months in patients achieving at least an MR (20%). In the phase I study [109] by Richardson et al. (C16003), MLN9708 was administered PO on days 1, 4, 8, 11 of 21 days’ cycles (upto 12 cycles). Twenty six patients were enrolled in the dose escalation cohort and received MLN9708 at doses of 0.24–2 mg/m^2^. MTD, determined to be 2 mg/m^2^ was administered to 34 patients of the four expansion cohorts: relapsed/refractory, BTZ-relapsed, PI-naïve and prior CFZ. The median age was 65 years (range 50–86 years) and the median number of prior therapies was four (range, 1–28); 88% patients had received BTZ, 88% LEN and 60% had undergone AHCT and 60% were refractory to the last therapy. The activity of twice weekly MLN9708 was similar to that reported by Kumar et al. using weekly dosing, and of the 55 evaluable patients, 15% achieved at least PR (16% at least MR) and 76% had at least SD. The most common G≥3 AE were thrombocytopenia and neutropenia occurring in 37% and 17%, respectively and 8% developed a manageable rash. No G3/4 PN was reported. Regarding PK, after day 11 dosing of MLN2238, t_1/2_ resulted to be 3.3–7.4 days, supporting twice weekly dosing schedule. Taking the two phase I studies together, 14%–21% of patients had AEs resulting in dose reductions and 6%–11% had to discontinue MLN9708. Only 10% reported PN, all G1–2, and all had PN at study entry. In a phase II study [110], 32 patients with two prior lines of therapy (range, 1–7), including IMiDs (88%), BTZ (28%) and AHCT (59%) received single-agent MLN9708 on days 1, 8 and 15 of 28-days cycle at a fixed oral dose of 5.5 mg, based on population PK analyses showing there was no impact of body size on MLN9708 exposure [111]. DEX (20 mg on the day of and the day after MLN9708) was added in patients who did not obtain at least MR after cycle 2, at least PR after cycle 4, or had progressive disease at any time. After four cycles of therapy at least MR and PR were achieved by 25% and 16% of patients, respectively, and 34% and 41% after adding DEX. No patient developed G3–4 PN and the main G3–4 AE, occurring in 56% patients, were thrombocytopenia, fatigue, nausea and diarrhea [112].

Impressed by the promising activity of single-agent MLN9708 in RRMM [108,109] and drawing from the efficacy of the BTZ-LEN-DEX combination [113], a phase I/II study [114] combining MLN9708 with LEN-DEX was conducted in NDMM patients (*n* = 65). The primary objectives of the phase I study were safety and tolerability and to establish MTD and the recommended phase II dose (RP2D) of the combination. The phase II study evaluated ORR and safety. Patients received MLN9708 (PO days 1, 8, 15), LEN (25 mg days 1–21) and DEX (40 mg weekly) for up to 12 cycles (28 days each). Induction therapy was followed by MLN9708 maintenance until progression. The phase I study enrolled 15 patients who were treated with MLN9708 at doses ranging from 1.68 to 3.95 mg/m^2^. MTD was found to be 2.97 mg/m^2^ and the RP2D was 2.23 mg/m^2^. Considering the balance between toxicity and efficacy across multiple cycles and PK, 4 mg fixed dose instead of body surface area-based dose was used for the phase II study [111] (*n* = 50). The median number of treatment cycles was seven (range, 1–27) and 38% received the maintenance. Toxicity included G3–4 neutropenia and thrombocytopenia in 12% and 8%, respectively, whereas the most common G3–4 non-hematologic side effects were skin rash (17%), fatigue (9%), vomiting and diarrhea (6% each). Thirty six percent patients developed PN, including G3 in 6% of them. ORR was 92% after four cycles (CR = 27%; VGPR = 35%). Deeper response was observed in patients receiving maintenance and all seven patients in CR evaluable for MRD by MPFC were confirmed to be MRD-negative. After a median of four induction cycles, 22 patients underwent AHCT. With a median follow-up of 14.3 months, 1-year OS was 94% [114]. The same all-oral combination, with MLN9708 given twice weekly, was investigated by Richardson et al. in a phase I/II study [115]. The phase I study enrolled 14 patients who received MLN9708 3 mg (*n* = 7) or 3.7 mg (*n* = 7) biweekly, LEN 25 mg on days 1–14 and DEX (20 mg; cycles 1–8, 10 mg; cycles 9–16) on days 1, 2, 4, 5, 8, 9, 11, 12 for up to sixteen 3-weekly cycles, after which MLN9708 maintenance was continued. Dose-limiting toxicity (DLT) was seen in the phase I study, and the RP2D was 3 mg. The median number of cycles administered to 64 patients (phase I = 14, phase II = 50) was nine (range, 1–30); 33% proceeded to AHCT and 14% discontinued treatment due to AEs. Among the 62 evaluable patients, ORR was 94% (including 26% ≥CR). Analysis of MRD by MPFC in patients with CR showed 75% were MRD-negative. There were no drug-related G4 AEs and most common G3 toxicities were rash (16%), pneumonia, thrombocytopenia, neutropenia and PN (~5% each) [115]. The higher rates of skin rash and dose reduction seen with this schedule supported the use of weekly dosing MLN9708 in phase III trials. TOURMALINE-MM1 study was a double-blind, placebo-controlled, phase III trial that randomized 722 RRMM patients to MLN9708 plus LEN-DEX (IRd) or placebo plus LEN-DEX (control group) [45]. The primary end point was PFS, which was significantly longer in the MLN9708 group than placebo at a median follow-up of 14.7 months (mPFS of 20.6 months vs. 14.7 months; HR 0.74, *p* = 0.01). PFS benefit was observed with MLN9708 in all pre-specified patient subgroups, including in patients with high-risk cytogenetic abnormalities. ORR was 78% with IRd and 72% in the control group (48% VGPR + 39% CR). The median DOR was 20.5 months and 15 months, respectively. At a median follow-up of 23 months, mOS had not been reached in either group. The rates of SAEs (47% in the MLN9708 group and 49% in the placebo), discontinuation of the study regimen because of AEs, and death during the study period (4% and 6%, respectively) were similar in the two groups. G ≥ 3 AEs occurred in 74% and 69% patients, respectively. Grade 3–4 thrombocytopenia occurred more frequently in the MLN9708 group (19%) than in the control group (9%). Rash (36% vs. 23%) and GI toxicity (predominantly low-grade) were more frequent with IRd. The incidence of PN was about similar (27% with MLN9708 vs. 22% with placebo; G3 in 2% of each group). MLN9708 (Ninlaro^®^, Takeda Pharmaceutical, Cambridge, MA, USA) was approved by FDA in 2015 based on the results of this study [116] in combination with LEN-DEX to treat MM patients who have received at least one prior therapy. This effective all-oral combination regimen (IRd) has generated significant interest and is now being investigated as upfront treatment for NDMM. 

A multicenter phase II study investigated another all-oral triplet of MLN9708, with oral Cy and low-dose DEX (ICd) as first-line therapy for transplant-ineligible NDMM patients [117]. Preliminary data demonstrated comparable activity across treatment arms with a manageable toxicity profile in line with previously mentioned MLN9708 studies. The study randomized 70 patients to MLN9708 (4 mg days 1, 8, 15) plus DEX 40 mg weekly and two different doses of Cy: 300 mg/m^2^ (ICd-300, *n* = 36) or 400 mg/m^2^ (ICd-400, *n* = 34) days 1, 8, 15 of 28-days cycle for up to 13 cycles. After a mean follow-up of 7 months, preliminary results showed ORR of 80% (ICd-300) and 73% (ICd-400), including CR + VGPR rates of 27% (ICd-300) and 23% (ICd-400). Toxicity was manageable but appeared increased with ICd-400. Most common (>15%) AEs included anemia, neutropenia, GI, fatigue and PN. Most common G≥3 AEs were neutropenia, anemia and pneumonia but no G3/4 PN was observed.

Maintenance treatment has become a mainstay of MM therapy. In the study by Kumar et al., 21 patients of the phase II study entered maintenance therapy with MLN9708, and 48% improved their response and no serious AEs were observed [118]. Based on the results of LEN maintenance post-AHCT showing a significant time to progression (TTP), PFS [119,120] and OS benefit [120], Shah et al. [121] studied the combination of MLN9708 with LEN as maintenance therapy post-AHCT in a phase II study. The primary objective was PFS while ORR, second primary malignancies (SPM), TTP and time to next treatment (TTNT) were secondary endpoints. Sixty to 180 days post-AHCT, 20 patients received maintenance LEN 10 mg daily continuously and MLN9708 4 mg on days 1, 8, 15 of 28 days cycle. Only one patient discontinued treatment due to AE (pneumonia) and six (30%) required dose reduction of one of the drugs. G3–4 non-hematologic toxicity was limited to G3 rash in one patient, whereas the most common hematologic AEs were neutropenia and thrombocytopenia (35 and 15%, respectively). Given the encouraging activity of MLN9708 in maintenance, two randomized phase III trials have been designed. TOURMALINE-MM3 is an ongoing study investigating MLN9708 vs. placebo as post-AHCT maintenance therapy, whereas transplant-ineligible NDMM patients are enrolled in TOURMALINE-MM4 study to investigate MLN9708 vs. placebo as maintenance therapy. If the results of these MLN9708 maintenance studies show promise in terms of improvement in PFS, then comparing MLN9708 with the established standard-of-care, LEN as maintenance therapy would be a future consideration.

### 4.3. Oprozomib

Oprozomib (ONX-0912, previously PR-047) [122] is a derivative of CFZ, an orally bioavailable peptide epoxyketone-based, irreversible PI that showed similar potency to CFZ in cytotoxicity assays [14,15,16]. Like BTZ and CFZ, ONX-0912 is highly selective for CT-L (β5) subunit of proteasome. Oral ONX-0912 showed equivalent antitumor activity to IV CFZ in animal models. It exhibits anti-myeloma activity through its anti-angiogenic and pro-apoptotic activity demonstrated on both in vitro and in vivo studies [123]. ONX-0912 results in activation of caspase-8, caspase-9, caspase-3 and PARP, and inhibits the migration of MM cells. It induces apoptosis in MM cells resistant to BTZ-based therapies. ONX-0912 is able to activate JNK and inhibit NF-κB pathways [124,125]. Further in vitro study showed that ONX-0912 inhibits growth, migration and induces apoptosis of MM cell lines and its activity is associated with activation of caspase-8, -9 and -3, and PARP. ONX-0912 potently induced apoptosis in tumor cell lines via upregulation of pro-apoptotic Bik and inhibited the growth of xenograft tumors in a dose-dependent manner [126]. Chauhan et al. [124] demonstrated that ONX-0912-treated mice had decreased tumor growth and angiogenesis. ONX-0912 also induced autophagy mediated, in part, by activation of the UPR pathway involving upregulation of ATF4 transcription factor. ONX-0912 promotes cell death in MM cells from patients who relapsed after treatment with BTZ, DEX or LEN [122].

A phase Ib/II single-agent open-label study of ONX-0912 by Vij et al. [127] was conducted in 106 patients, of whom 68 had MM that had relapsed after more than one prior therapy, to determine the MTD and safety profile. ONX-0912 was administered once daily on days 1, 2, 8 and 9 of a 14-day cycle (schedule 2/7) or on days 1–5 of a 14-day cycle (schedule 5/14). The initial dose was 150 mg/d and was escalated in 30 mg increments to a maximum of 330 mg/d. The RP2D was set at 240 mg/d days 1–5 of a 14-day cycle. An ORR of 35% was seen in CFZ-naive patients in phase Ib, whereas in BTZ-refractory patients, ORR was 14%. In phase II, ORR was 27% in CFZ-refractory patients (*n* = 11), 33% in CFZ-sensitive patients (*n* = 12) and 25% in BTZ-refractory patients (*n* = 12). A dose of 300 mg/d in schedule 2/7 and 240 mg/d in schedule 5/14 were established as MTD. DLT of ONX-0912 were hypotension, diarrhea and thrombocytopenia. The most common AEs of ONX-0912 were anemia, thrombocytopenia, nausea, vomiting and diarrhea. Hari et al. [128] tested ONX-0912 plus DEX in a multicenter phase Ib/II study to evaluate the safety and tolerability in 29 RRMM patients who had received at least one prior therapy including LEN and/or BTZ. ONX-0912 doses started at 210 mg/d and were administered on the same schedule as in the aforementioned study: schedule 2/7 or schedule 5/14, with 30 mg/d dose increments. DEX was given on days 1, 2, 8 and 9 of a 14 days cycle. Fourteen patients on schedule 2/7 had no DLT. However, three DLT were observed in the 15 patients on schedule 5/14 (G2 subarachnoid hemorrhage, G3 transaminitis and G4 thrombocytopenia). PR was achieved in five of 12 evaluable patients (schedule 2/7). No PR was seen among seven evaluable patients on the schedule 5/14. 

Another phase Ib/II trial was conducted to assess the safety and efficacy of the combination of ONX-0912 with POM-DEX in 31 RRMM patients who had failed BTZ and either LEN or THAL, using the following dosing regimen: ONX-0912 150 mg/d PO (schedule 5/14, *n* = 4) or 210 mg/d (schedule 2/7, *n* = 17) of 28 days cycle with subsequent escalations, POM 4 mg PO days 1–21 and DEX 20 mg orally on days 1, 2, 8, 9, 15, 16, 22 and 23. MTD of ONX-0912 was 210 mg/d. ORR was 50% (schedule 5/14 cohort) and 59% (schedule 2/7 cohort). Significant benefit was observed from the combination which was tolerated with minimal side effects [129]. A phase III study comparing the combination of ONX-0912 and POM-DEX with placebo and POM-DEX is currently ongoing and will look at several response and survival endpoints [130]. 

### 4.4. Marizomib

Marizomib (NPI-0052) is an irreversible PI with a β-lactone backbone and the only non-peptide-based PI that was isolated from the marine actinomycete *Salinispora tropica* [131]. Being a non-peptidic PI, NPI-0052 is expected to have better bioavailability than other PIs that are peptide-mimetics and so can be degraded by endogenous proteases and peptidases in the plasma [106]. It differs structurally from other PIs and these structural differences translate into significant differences in proteasome inhibition, toxicity and efficacy profiles [132,133]. NPI-0052, which also has good oral bioavailability, is an inhibitor of all three 20S proteasome catalytic subunits [17]: it binds irreversibly with high affinity to the CT-L (β5) and T-L (β2) catalytic sites as well as with lower affinity to the C-L (β1) subunit [133]. NPI-0052 inhibits the canonical NF-κB pathway and related secretion pathways, such as interleukin-6 (IL-6), TNF-α and IL-1b [133]. Unlike BTZ, which activates both caspase-8 and -9, the apoptotic effect of NPI-0052 is mainly mediated by caspase-8 activation and to a lesser extent, by caspase-9 [30]. This mechanism of activation of caspases through caspase-8 allows overcoming the resistance of MM cells with mutations causing overexpression of Bcl-2. It has been shown that Bcl-2 overexpression confers resistance and partially protects MM cells against BTZ. However, caspase-9 activation by NPI-0052 is minimally affected by Bcl-2 overexpression. Apoptotic signal leads to the release of cytochrome c and Smac proteins from mitochondria to the cytoplasm, generation of oxygen radicals and activation of caspases. Moreover, NPI-0052 is able to induce apoptosis in MM cells even in the presence of MM growth factors IL-6 and insulin growth factor-1 (IGF-1) and is involved in blocking IL-6 secretion in bone marrow stromal cells (BMSC) without affecting their viability. NPI-0052 also significantly blocks MM cells migration induced by VEGF and thus confirms its anti-angiogenic effect [133]. Preclinical studies have shown NPI-0052 induces apoptosis of MM cells, including cells resistant to conventional therapies as well as cells isolated from patients who had developed resistance to BTZ, but did not affect normal lymphocyte viability, viability of MM patient-derived BMSC [133,134,135] or cause significant toxicity to other hematopoietic cell lines or normal human neural stem cells. In addition, synergistic in vitro and in vivo activity was seen when NPI-0052 was tested in combination with BTZ. This is in part explained by the fact that NPI-0052 and BTZ trigger differential apoptotic signaling pathways and partly by the irreversible pattern of proteasomal inhibition by NPI-0052 [136]. In vivo, NPI-0052 has shown prolonged survival in animal models of MM [133]. A human MM xenograft model in mice reduced MM tumor growth and prolonged survival after twice weekly IV or oral administration of NPI-0052. NPI-0052 has exhibited synergistic activities in tumor models in combination with BTZ, LEN, and HDACi [17,136,137,138,139,140]. NPI-0052 exhibits an extremely short half-life (<5 min) and wide tissue distribution (including penetration of blood-brain barrier).

Although it is orally active, the trials of NPI-0052 performed to date have used IV formulation. NPI-0052-101, a phase I study, enrolled RRMM patients in a dose-escalation design to determine the MTD and RP2D of NPI-0052 on two schedules [141]: schedule A (0.025–0.7 mg/m^2^ IV weekly on days 1, 8, and 15 of 4-week cycles) and schedule B (0.15–0.6 mg/m^2^ IV on days 1, 4, 8, and 11 of 3-weekly cycles; DEX was allowed with schedule B). A total of 32 patients received schedule A and RP2D was 0.7 mg/m^2^ infused over 10 min, whereas 36 patients received Schedule B and RP2D was 0.5 mg/m^2^ infused over 2 h. The most common (>20%) drug-related AEs were fatigue, headache, nausea, diarrhea, dizziness, and vomiting. Five patients had PR (1 patient on schedule A and 4 on schedule B; 3 of the 4 patients also received DEX). NPI-0052-102 was a phase I study evaluating MTD, PK, and PD of NPI-0052 IV on two dosing schedules in patients with advanced malignancies [142]: 42 patients received schedule A (0.1–0.9 mg/m^2^ IV over 1–10 min on days 1, 8, 15 in 4-weekly cycles) and 44 patients with RRMM (80%) and other hematologic malignancies received schedule B (0.075–0.6 mg/m^2^ over 1 min to 2 h on days 1, 4, 8, 11, in 3-weekly cycles). RP2D for schedule A was 0.7 mg/m^2^ over 10 min and for schedule B, 0.5 mg/m^2^ over 2 h. The most common (>25%) drug-related AEs were fatigue, nausea, diarrhea, and infusion site pain (schedule A), and fatigue (schedule B); most were G1–2. ORR of 11% was seen in 27 evaluable RRMM schedule B patients (1 VGPR, 3 PR, 4 MR, and 12 SD). No significant PN or thrombocytopenia was observed. DLT included cognitive changes, expressive aphasia, visual/auditory hallucinations, disorientation and unsteady gait, which were reversible. From preclinical studies, marizomib is known to cross the blood-brain barrier, and therefore, IV infusion time was increased to 2 h to ameliorate central nervous system (CNS) toxicities observed with 10 min infusion that were thought to be related to the maximum concentration that occurs at the end of the infusion. Both -101 and -102 trials showed that NPI-0052 has activity in heavily-pretreated RRMM patients. Clinical responses to NPI-0052 have been observed in patients with BTZ-refractory MM and its safety profile differs from BTZ, with no significant treatment-emergent PN or myelosuppression reported, but CNS penetration accounts for the unique neurologic DLT. 

Spencer et al. [143] enrolled 38 patients into a dose-escalation phase I combination trial of NPI-0052 with POM-DEX to assess safety, PK and efficacy of the regimen. Patients had received multiple prior regimens (median 4, range 1–9), including both LEN and BTZ, and were refractory to their last therapy. A third previously received CFZ and half received THAL; 53% were refractory to BTZ and LEN. NPI-0052 was given IV at 0.3–0.5 mg/m^2^ over 2 h on days 1, 4, 8 and 11 in combination with POM-DEX in 4-weekly cycles. ORR for the 36 evaluable patients was 53%. Since no DLT was observed, MTD was not exceeded, and the highest dose cohort, NPI-0092 0.5 mg/m^2^, POM 4 mg and DEX 10 mg, was established as the RP2D. The most common treatment related ≥G3 AEs were neutropenia (29%), pneumonia (11%), anemia (11%), thrombocytopenia (11%), and febrile neutropenia (5%), with two G4 AEs (neutropenia and viral infection), and one G5 AE (cardio-respiratory arrest from a suspected PE related to POM). Overall, NPI-0052 was well tolerated, did not add to the incidence or severity of POM-DEX AEs. PK analysis revealed it has a short t_1/2_ (6.2–11 min) and a large volume of distribution (41–86 L) suggesting extensive tissue distribution. NPI-0052 has shown activity as a single agent as well as in combination in patients with RRMM. As it appears to have activity in BTZ-refractory patients, it warrants further investigation. 

Given the unique property of CNS penetration, NPI-0052 has been used to treat 3 patients (under compassionate use protocol) for CNS-MM [144]. Two of the 3 patients were evaluable for the activity of NPI-0052 plus DEX. NPI-0052 was dosed (0.5–0.7 mg/m^2^ as 10 min IV infusion days 1, 8, 15 of 28 days cycle). Both patients, who had no response to prior craniospinal radiation and intrathecal chemotherapy, tolerated NPI-0052 with no unexpected AEs. One had an 89% reduction in cerebrospinal fluid (CSF) plasmacytosis and 77% serum lactate dehydrogenase (LDH) reduction. After 5 months of treatment, daratumumab was added to NPI-0052 and further clinical improvement was observed, i.e., resolution of diplopia and regaining full ambulation. PFS was 6 months. In the other patient, symptom resolution accompanied by complete resolution of CSF plasmacytosis and CSF M-spike occurred in the second month of treatment with NPI-0052. With the eighth cycle, POM was added to the regimen, though the patient had not progressed. These anecdotal cases provide preliminary evidence of therapeutic role of NPI-0052 in CNS-MM.

## 5. Histone Deacetylase Inhibitors

### 5.1. Histone Deacetylases: A Therapeutic Target

Epigenetic processes are a means of affecting gene expression without altering the nucleic acid (DNA) sequence [145,146,147,148]. Dysregulated HDAC activity is an epigenetic hallmark of MM, resulting in aberrant gene expression and cellular signaling that promotes cell growth and survival, and resistance to apoptosis [149,150,151]. Acetylation and deacetylation of histones catalyzed by histone acetyl transferases (HAT) and HDAC are one of the fundamental modification processes of biologic significance [148]. In general, hyper-acetylated chromatin is transcriptionally active, and hypo-acetylated or deacetylated chromatin is transcriptionally silent [116]. Transcriptional machinery is unable to access DNA when chromatin is condensed secondary to the removal of acetyl groups on core histones [152]. Altering the acetylation of chromatin may thus alter the expression of oncogenes and tumor suppressors and that influences oncogenesis. In addition, specific DNA residues may be deacetylated, altering the binding of transcription factors. This may enhance or repress transcription altogether [152].

**Figure 5:**
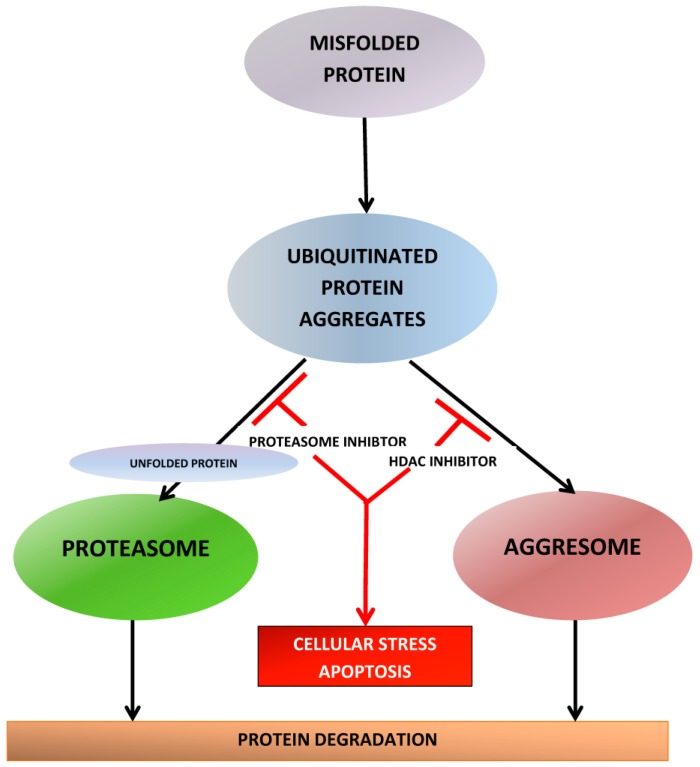
Inhibitions of the proteasome and aggresome pathways. Misfolded proteins are ubiquitinated for degradation by the proteasome and aggresome pathways. The proteasome inhibitors lead to the accumulation of ubiquitin-protein aggregates. These aggregates are transported to the lysosome, where they are degraded by the aggresome pathway. The interaction of the unfolded and/or misfolded protein complexes is facilitated by histone deacetylase 6 (HDAC6) and HDACi blocks this process. The combination of proteasome inhibitors (PI) and histone deacetylase inhibitor (HDACi) leads to increased cellular stress and apoptosis. Adapted from Hideshima et al. [153].

There are several thousands of acetylation sites on proteins associated with various intracellular functions including gene expression, DNA replication and repair, cell-cycle progression, cytoskeletal reorganization, and protein chaperone activity. Therefore, besides the effect on the acetylation status of histones, HDAC inhibition also affects other cellular processes and can lead to a variety of biologic effects downstream important for cellular proliferation, angiogenesis, differentiation and survival. A total of 18 HDACs have been identified and grouped into four classes based on their homology to yeast HDACs, subcellular localization, and enzymatic activities [154,155,156]. The classes differ in tissue expression and protein targets [157]. Class I HDACs include HDAC1-HDAC3 and HDAC8, which are localized to the nucleus and primarily act on histone proteins and transcription factors [158]. Class II HDACs include HDAC4-HDAC7, HDAC9, and HDAC10 [158]. They are thought to move between the nucleus and cytoplasm, where they act primarily on non-histone proteins. HDAC6 plays an essential role in protein degradation via the aggresome pathway [159]. HDAC6 binds to polyubiquitinated misfolded proteins and recruits them for transport to aggresomes, which are transported by microtubules to an autophagosome, where they are degraded by autophagy. This “rescue” pathway is vital to MM cells that produce a heavy burden of misfolded proteins and may have overwhelmed UPS pathway. The blockage of UPS does not completely eliminate metabolism of misfolded proteins because of the presence of the aggresome, which acts as a backup, and enables MM cells to escape the lethality of proteasome inhibition [160] and eventually leads to development of PI resistance. HDAC6 inhibition blocks aggresome formation, thereby inhibiting the degradation of and causing accumulation of misfolded proteins within cells. This complements proteasome inhibition and explains the synergy between HDACi and PIs [161] (Figure 5). 

### 5.2. Histone Deacetylase Inhibitors: Mechanisms of Anti-Myeloma Activity

HDAC inhibition has been described as a master switch that could simultaneously affect multiple pathways critical for the survival of MM cells. As a class, HDACi inhibit the actions of HDAC enzymes and affect the expression of genes that regulate of cancer cell survival via a number of mechanisms. HDACi bind to the catalytic domains of HDACs, downregulating their activity, which in turn inhibits MM cell survival and proliferation [20]. Most HDACi arrest the cell cycle at G1-M phase [162] and induce apoptosis by upregulation of many proapoptotic proteins and downregulation of antiapoptotic proteins such as Bcl-2 [163]. In addition, HDACi have a number of direct and indirect effects that contribute to oxidative damage to cellular DNA. They cause delays in mitosis by overcoming the spindle assembly checkpoint through changes in tubulin [164]. HDACi also inhibit hsp90, a cellular chaperone required for proteins involved in intracellular signaling (Raf, Her2/neu, ERK, NF-κB) [165]. HDACi also exhibit antiangiogenic effects and induce autophagy [164], cause acetylation of tubulin and disruption of aggresome formation and affect tumor immunity via effects on T cell receptor function, cytokine milieu of immune effector cells, and direct upregulation of proteins on malignant cells that enhance cellular recognition by antigen presenting cells and other immune effectors. Many of these mechanisms also lend to direct synergy in the treatment of MM when used in combination with corticosteroids or PIs [153]. Proteasome inhibition in combination with aggresome inhibition by HDACi leads to cellular accumulation of proteins and hyperacetylation of tubulin, leading to apoptosis [153,165]. Transcription factor NF-κB translocates into the nucleus, and promotes cell survival with transcription of various genes such as pro-inflammatory cytokines and anti-apoptotic proteins such as Bcl-2 [166]. Inactivation of NF-κB by deacetylase inhibition and proteasome inhibition result in synergistic apoptotic activity. Finally, HDAC inhibition allows for the expression of numerous tumor suppressor genes. Combination therapy with PI allows for a decreased breakdown of tumor suppressor proteins. Class I HDACi acetylate histone lysine residues, opening chromatin for protein synthesis and gene expression. HDACi that non-selectively inhibit a broad range of HDAC (pan-HDACi) [158] have been studied in MM and include panobinostat (PANO) and vorinostat (SAHA) [47,48,158,167]. HDACi that selectively target HDAC6 (HDAC6i), such as ricolinostat (ACY-1215) and ACY-241, increase acetylation of tubulin and disrupt transportation of aggresomes, and are being investigated for MM treatment [153,168]. Both preclinical and clinical data has supported the use of HDACI in combination with other agents, most strikingly with PI. Besides targeting the UPS and NF-κB, BTZ may target HDAC and may function as HDACi as well, further strengthening the rationale to use it in combination with HDACi [166]. Kikuchi et al. [169] reported that BTZ can downregulate the expression of class I HDAC (HDAC1, HDAC2, and HDAC3) in MM cell lines at the transcriptional level accompanied by histone hyperacetylation. Short interfering RNA-mediated knockdown of HDAC1 enhanced BTZ-induced apoptosis, whereas overexpression of HDAC1 conferred resistance to BTZ in MM cells and administration of HDACi romidepsin restored sensitivity of HDAC-overexpressing cells to BTZ. Pei et al. [170] first showed that, in vitro, the combination of HDACi with BTZ resulted in enhanced cellular killing compared with their effects as single agents. This synergy was associated with a reduction of NF-κB DNA binding activity, modulation of JNK activation, and a ROS-dependent downregulation of cyclin D1, Mcl-1, and XIAP. Inhibition of aggresomal pathway by tubacin, together with proteasomal inhibition by BTZ, also resulted in an accumulation of ubiquitinated proteins followed by synergistic anti-MM activity [165].

## 6. Clinical Development of Histone Deacetylase Inhibitors

### 6.1. Vorinostat

Evidence that HDACi induce apoptosis in human MM cells in vitro prompted investigation of these agents in RRMM [171,172,173]. Vorinostat (also known as suberoylanilide hydroxamic acid; SAHA) is an orally bioavailable, non-specific HDACi, is the prototype of a series of hydroxamic-acid-based HDACi that has been shown to induce MM cell apoptosis, with increased p21 and p53 protein levels and dephosphorylation of Rb, sensitize MM cells to other agents and inhibit the secretion of IL-6 triggered by MM cell binding to BMSC, suggesting that SAHA can overcome cell-adhesion mediated drug-resistance [174]. Further studies have shown that MM cells treated with SAHA exhibit myriad anti-proliferative and pro-apoptotic molecular events, including downregulation of transcripts for the IGF/IGF-1 receptor and IL-6 receptor signaling cascades, anti-apoptotic molecules (e.g., caspase inhibitors), oncogenic kinases, DNA synthesis/repair enzymes, and transcription factors (e.g., XBP-1, E2F-1) implicated in myeloma pathophysiology [3]. Importantly, SAHA suppresses the activity of the proteasome and enhances MM cell sensitivity to BTZ [171].

Based on the preclinical studies, a phase I dose-escalation study of oral SAHA (200, 250 and 300 mg PO BID for 5 days/week/4-weekly cycle or 200, 300, or 400 mg twice daily for 14 days/3-weekly cycle) enrolled 13 patients with RRMM to assess PK, safety and efficacy [175]. AEs (fatigue, nausea, diarrhea, dehydration) were manageable (mostly G1–2) and, importantly, no significant myelosuppression or PN was noted. Of 10 evaluable patients, one MR and nine SD were observed. Though having modest single-agent activity, SAHA seemed to improve outcomes in BTZ-refractory MM patients, given in combination with BTZ [176]. This combination was evaluated in two multicenter phase I studies [148,177,178]. In the first trial, 34 patients with RRMM were enrolled and received escalating doses of SAHA (200 mg BID, 300–400 mg daily for 4 days) and BTZ (0.7–1.3 mg/m^2^ IV on days 1, 4, 8, and 11) in 21 day cycles [179]. Eighteen had prior BTZ exposure and of these, 7 were BTZ-refractory. The highest doses of SAHA (400 mg daily for 14 days) and BTZ (1.3 mg/m^2^) were given together, but MTD was not reached. Nausea, vomiting, diarrhea, thrombocytopenia were the most common AEs (60%–74%, 9% G ≥ 3 each). PR was achieved in 9 (27%) patients, while 2 (6%) had MR and 20 (59%) had SD. Six BTZ-refractory patients had SD, while one had PR. The second phase I study enrolled 23 patients who received SAHA (100–500 mg on days 4–11) and BTZ (1–1.3 mg/m^2^ on days 1, 4, 8, and 11) in 21 days cycles [180]. Patients had received a median of seven prior regimens (range, 3–13), including BTZ in 19, of whom nine were BTZ-refractory. The toxicity was primarily hematologic. The most common G3–4 AEs were cytopenias (*n* = 13), fatigue (*n* = 11) and diarrhea (*n* = 5). Two patients receiving 500 mg/d of SAHA had QTc prolongation and fatigue as DLT. ORR was 42%, including three PR in BTZ-refractory patients. 

VANTAGE 095 study demonstrated that the combination of SAHA and BTZ was active in patients with MM [46]. This was an open-label, single-arm, multicenter, phase IIb study designed to evaluate the efficacy and tolerability of SAHA (400 mg/d days 1–14) combined with BTZ (1.3 mg/m^2^ IV days 1, 4, 8, and 11) in BTZ-refractory RRMM patients who had received an IMiD-based regimen. Patients received 21 days cycles of BTZ and SAHA. A total of 143 patients were enrolled in the trial. DEX, 20 mg, on the day of and the day after each dose of BTZ could be added for patients with progressive disease or no response. ORR was 11.3%, and the median DOR was 211 days. The mOS was 11.2 months, with a 2-year OS of 32%. The frequently reported AEs (>50%) were thrombocytopenia, nausea, diarrhea, anemia; the safety profile was consistent with that of BTZ and SAHA. Although this study did not meet its primary efficacy endpoint, the results confirmed the feasibility of the combination. VANTAGE 088 [47] was a randomized, double-blind, placebo-controlled phase III trial evaluating the efficacy and tolerability of SAHA in combination with BTZ to BTZ-placebo for treatment of patients with relapsed MM [47]. Patients who relapsed after at least one prior therapy and were not BTZ-refractory were enrolled and given 21 days cycles of BTZ (1.3 mg/m^2^ IV on days 1, 4, 8, and 11) in combination with oral SAHA (400 mg) or placebo daily on days 1–14. The primary endpoint was PFS. A total of 637 patients were enrolled (317 to the SAHA + BTZ and 320 to BTZ only). With a median follow-up of 14 months, mPFS were 7.6 months and 6.8 months (HR 0.77, *p* = 0.01), respectively. Although the ORR was improved with the SAHA + BTZ combination (56% vs. 40%), CBR (ORR + SD) was not much different (81% vs. 78%). The most common G3–4 AEs in the SAHA and control groups were thrombocytopenia (45% vs. 24%), neutropenia (28% vs. 25%), and anemia (17% vs. 13%). The 0.8-month PFS benefit is not considered clinically meaningful, and suggests that the responses in the SAHA arm were not durable [158]. The efficacy of this combination proved lower than expected which could be explained in part by the increased toxicity with concurrent use of PI and HDACi. Additionally, more patients in the SAHA arm had G3–4 AEs, including thrombocytopenia, fatigue, gastrointestinal (GI) symptoms. It is possible that different treatment schedules, different route of BTZ administration (i.e., SQ) or the addition of DEX may modify tolerability and/or efficacy of the combination [158].

A phase I dose-escalation study of SAHA in combination with LEN-DEX in RRMM patients was conducted to determine the MTD, in addition to safety and efficacy. MTD was not reached. Patients tolerated the highest administered dose level in the study (400 mg SAHA on days 1–7 and days 15–21, 25 mg LEN on days 1–21 and 40mg DEX on days 1, 8, 15 and 22), which was considered RP2D. The combination was generally well tolerated and of 30 patients evaluable for efficacy, 47% achieved ≥PR [181]. ORRs were lower among patients relapsed/refractory to prior LEN (10%, *n* = 10) or PI (15%, *n* = 13). Common AEs included anemia (58%), diarrhea (55%), fatigue (55%), and cough (45%), which were generally manageable. Subsequently, a phase IIb combination study evaluated the efficacy of this combination in LEN-refractory patients [182]. Patients received oral SAHA 400 mg days 1–7 and 15–21, LEN 25 mg days 1–21, and DEX 40 mg days 1, 8, 15 and 22 in 28-days cycles. Twenty-five patients were enrolled with median age of 65 years and median of 5 prior regimens. ORR was 24% (6 PR) and CBR (≥SD) was 80%. Median DOR was 3.3 months and mPFS was 5.3 months. Most common G3/4 AEs were neutropenia (48%), thrombocytopenia (32%), anemia (20%) and GI toxicities (16%). Vesole et al. reported the results of phase I, dose-escalation study investigating the tolerability and activity of a quadruplet regimen of CFZ, LEN, SAHA and DEX for the treatment of RRMM patients [183]. Seventeen patients received CFZ (15, 20, or 20/27 mg/m^2^; days 1, 2, 8, 9, 15, 16), LEN (15 or 25 mg; days 1–21), SAHA (300 or 400 mg; days 1–7, 15–21), and DEX (40 mg; days 1, 8, 15, 22) in 28 days cycles. No DLT was observed and MTD was not reached. G3–4 AEs were more common than the KRd regimen, and included neutropenia (53%), thrombocytopenia (53%) and anemia (41%), but none discontinued treatment because of a treatment-related AE. In this heavily pre-treated patient population (median of 4 prior therapies; a third were refractory to BTZ and LEN), ORR was 53% (12% VGPR and 41% PR). At a median follow-up of 10 months, mPFS was 12 months while mOS was not reached. In summary, the early enthusiasm about vorinostat has turned into loss of interest and no new trials as the results of the combination trials have not demonstrated significant improvement in clinical outcomes, but showed that SAHA did add to the toxicity of the regimens. The focus of clinical investigation has shifted toward another potent HDACi, panobinostat and selective HDAC6 inhibitors.

### 6.2. Panobinostat

Panobinostat (PANO) is a cinnamic hydroxamic acid HDACi with highly potent inhibitory activity for class I, II, and IV HDAC [184]. Preclinical studies showed that PANO has anti-myeloma activity [153] that relates to modifications to intracellular activity that alter the MM cell interactions with its microenvironment. PANO mediates upregulation of p21, leading to cell cycle arrest and apoptosis and interruption of the signaling pathway between the myeloma cells and the microenvironment which comprises the BMSC and extracellular matrix [174]. Consequently, clinical trials against MM followed: a phase I/II dose-escalation trial of oral PANO was conducted in 176 patients with hematologic malignancies, including 12 patients with RRMM [185]. Two dose-escalation regimens were evaluated (TIW or BIW). PANO doses ranged from 20 to 80 mg. RP2D for PANO was 40 mg TIW given weekly, and MTD was 60 mg BIW. One RRMM patient had a PR. AEs included GI and hematologic. Overall, this trial demonstrated safety and informed dosing for the subsequent monotherapy and combination therapy studies. A phase II trial conducted by Wolf et al. [186] evaluated PANO as monotherapy in 38 patients with RRMM using PANO 20 mg dose TIW given weekly in 21 days cycles. Patients had received at least two prior lines of therapy including both an IMiD and BTZ. Overall activity was limited, with one PR and one MR (DOR of 19 and 28 months, respectively). Over 80% of patients had GI AEs, the majority of which were G1–2. Most G3–4 AEs were hematologic AEs (cytopenias). The trial was closed for insufficient efficacy.

Considering the low single-agent activity of PANO in MM, manageable toxicity profile and preclinical data on potential synergy of HDACi with BTZ-DEX, combination trials ensued [153,187,188]. A phase Ib trial was conducted in RRMM patients (*n* = 62) to determine MTD of PANO in combination with BTZ-DEX [189]. In the dose-escalation phase, 47 patients received PANO starting at 10 mg TIW given weekly, in combination with BTZ starting at 1 mg/m^2^ on days 1, 4, 8, and 11 of a 21 days cycle ± DEX 20 mg on the days of and after BTZ. The dose-expansion phase involved 15 patients receiving PANO 20 mg TIW for weeks 1 and 2, BTZ 1.3 mg/m^2^ and DEX 20 mg using the same dosing schedule as before. ORR in the escalation phase was 45% (all patients) and 53% in those who received the MTD. Twenty percent obtained ≥VGPR. Median DOR was 138 days. ORR in the expansion phase was 73% (26% for BTZ-refractory), with mDOR of 159 days. G3–4 AEs were mostly hematologic: thrombocytopenia (85% and 67%), neutropenia (64% and 47%), and asthenia/fatigue (30% and 20%) in the escalation and expansion phases, respectively. Based on the promising phase Ib trial, the PANORAMA program was designed to further investigate the anti-myeloma activity and safety of PANO-BTZ-DEX regimen [190]. PANORAMA 2 was a single-arm, two-stage phase II study in RRMM patients based on the MTD derived from the phase Ib. The study enrolled 55 heavily pretreated BTZ-refractory MM patients who had received at least two (median, 4) prior lines of therapy, including IMiD [191]. In the first phase, patients were treated for up to eight 3-weekly cycles of PANO 20 mg TIW for weeks 1 and 2, BTZ 1.3 mg/m^2^ days 1, 4, 8, 11 and DEX 20 mg/d. Patients with clinical benefit (*n* = 17) advanced to the second phase, and received 6-weekly cycles of PANO 20 mg TIW for weeks 1, 2, 4, and 5; BTZ 1.3 mg/m^2^ weekly in weeks 1, 2, 4, and 5; and DEX 20 mg. ORR was seen in 35%, while clinical benefit (≥MR) was seen in 53%, but no patient achieved CR. These results suggested that PANO was capable of recapturing responses to BTZ in BTZ-refractory patients [191]. The most common G3–4 AEs included thrombocytopenia (64%), diarrhea (20%), fatigue (20%). Median PFS was 5.4 months and mOS of 17.5 months. Responses were durable with mDOR of 6 months. A randomized, double-blind phase III trial (PANORAMA 1) of RRMM patients followed that compared PANO-BTZ-DEX (*n* = 387) with placebo-BTZ-DEX (*n* = 381). BTZ-refractory patients were excluded [48]. The study consisted of two treatment phases as in PANORAMA 2. Patients had received a median of one prior line of therapy (range, 1–3). The addition of PANO to BTZ-DEX significantly improved PFS (which was the primary end-point) by 4 months (mPFS of 12 months vs. 8.1 months; HR 0.63, *p* < 0.0001). This benefit was maintained regardless of age, ISS stage, prior lines of therapy or prior treatment with BTZ or IMiDs. ORR was not significantly increased with PANO (61% vs. 55%; *p* = 0.09), but deep responses (CR + nCR) were more frequent with PANO (28% vs. 16%; *p* = 0.00006). OS in the PANO arm was 40.3 months and 35.8 months in the control arm, but this difference was not statistically significant (HR 0.94; 95% CI, 0.78–1.14). Of note, a higher percentage of patients in the placebo arm received post-study therapy, which may have confounded the OS results [49]. PANO cohort was almost twice as likely to have G3–4 thrombocytopenia, neutropenia, diarrhea, fatigue. Higher rate of discontinuation due to AEs was seen with PANO (36% vs. 20%) [48]. Pre-planned subgroup analyses revealed the benefit with PANO was greatest in those who received two or more prior therapies (*n* = 147) including BTZ and IMiDs [192]. The PFS benefit was 7.8 months with PANO-BTZ-DEX (12.5 months vs. 4.7 months; HR 0.47). OS was, again, not significantly different between the two subgroups (25.5 months vs. 19.5 months, respectively; HR 1.01) [192]. Sub-analyses also indicated that those who were able to tolerate and receive a longer duration of treatment with PANO-BTZ-DEX also had a longer PFS. Those who completed the first phase treatment had a mPFS of 14.6 months, compared to 17.6 months for those who completed the second phase as well [49]. Based on the strength of PANORAMA 1 results, particularly the subgroup analysis, FDA, in 2015, approved PANO-BTZ-DEX regimen for the treatment of RRMM patients who have received ≥2 prior regimens including BTZ and IMiD [116]. 

A variety of novel combination strategies have also been explored, including combinations of PANO with novel PIs and IMiDs. Several phase I/II studies have investigated PANO in combination with CFZ or CFZ-DEX for RRMM. A phase I/II dose-escalation and expansion trial in RRMM patients (*n* = 80) was conducted evaluating PANO-CFZ combination [193]. In the dose-escalation portion of the trial, MTD was not established, but the planned expansion dose was established as Dose Level 4 (DL4; PANO 30 mg on days 1, 3, 5, 15, 17, 19 and CFZ 20/45 mg/m^2^ IV on days 1, 2, 8, 9, 15, and 16; 28 days cycle). The expansion phase was modified to include Dose Level 6 (DL6) using PANO 20 mg and CFZ 20/56 mg/m^2^ doses given on the same schedule, besides DL4 (*n* = 32 + 32) [194]. Preliminary results indicated an ORR of 75% for all patients; 72% for DL4 (40% ≥ VGPR) vs. 84% for DL6 (37% ≥ VGPR). The mPFS was ~8.6 months (both DLs). Median OS was 28.2 months for DL4 and not reached for DL6. There were no significant differences in AEs between the two dosing levels. The most common AEs (>50%) were thrombocytopenia, nausea, diarrhea and fatigue/asthenia. Of note, G3 GI AEs (none G4) occurred in 3%–9% patients, G3–4 thrombocytopenia in 42% and G3–4 cardiac events in 6%, but none developed G3–4 PN. Both expansion dose levels demonstrated efficacy and were well-tolerated; in fact, safety profile seemed favorable compared to PANORAMA 1 trial. An increase in CFZ dose from 45 mg/m^2^ to 56 mg/m^2^ between the two expansion DLs did not increase cardiac toxicity, whereas decreasing PANO dose from 30 mg to 20 mg appeared to reduce low-grade GI toxicity, supporting further development of DL6.

Shah et al. conducted a study evaluating PANO 15–30 mg on days 1, 3, 5, 8, 10, 12 and CFZ (20 mg/m^2^ IV on days 1 and 2 of cycle 1; 27 mg/m^2^ on days 8, 9, 15, 16 of cycle 1; and 27–45 mg/m^2^ ≥cycles 2) and DEX (4 mg on CFZ days) in 28 days cycles in RRMM patients [195]. The Phase I study enrolled 21 patients who had received a median of 5 prior therapies. There was 1 DLT (G4 thrombocytopenia with epistaxis) with PANO 20 mg plus CFZ 20/36 mg/m^2^. Common G3–4 AEs included thrombocytopenia (52%), anemia (43%), neutropenia (29%), fatigue (19%), lung infection/pneumonia (19%), elevated creatinine (14%), diarrhea (10%). The ORR was 29%, including 10% VGPR. The recommended expansion phase dose was PANO 20 mg plus CFZ 20/45 mg/m^2^. A phase I trial conducted by Kaufman et al., is investigating PANO 15–20 mg with CFZ-DEX in 26 RRMM patients, [196] who had received a median of 3 prior lines. The MTD was determined to be PANO 20 mg plus CFZ 20/36 mg/m^2^. Common G3–4 AEs were mostly hematologic, consistent with other studies. The ORR was 46%, including 23% ≥VGPR. Median DOR was 7.5 months and mPFS was 11.4 months. Among BTZ-refractory patients, ORR was 44%. A phase I trial investigated the combination of PANO with MLN9708-DEX in heavily-pretreated RRMM patients (median of five prior regimens) [197]. PR rate was 9% (1 of 11) and 18% had MR (2 of 11). No non-hematologic G3–4 toxicity was observed, and three patients developed G3 hematologic AEs. No SAE was observed and no dose reduction of PANO or MLN9708 was required. These results suggest that the combination is well-tolerated and further study is needed to assess the efficacy of this combination. The RP2D was established at PANO 20 mg TIW weeks 1 and 3, MLN9708 4 mg days 1, 8, 15 and DEX 20 mg on the day of and after MLN9708.

Akin to PIs, preclinical synergistic anti-myeloma activity has been demonstrated with the addition of PANO to an IMiD (LEN). HDACi combined with IMiD has synergistic cytotoxicity of MM cell lines via downregulation of MYC expression as well as caspase activation and down-regulation of anti-apoptotic factors [198]. A combination of PANO, LEN and DEX for treatment of mice with MM1S plasmacytomas slowed tumor growth and prolonged survival in the mice better than the combination of any of the two individual agents [187]. A phase I/II clinical trial of PANO combined with LEN-DEX was conducted in RRMM patients with at least one prior line of therapy [194]. The phase I identified RP2D at PANO 20 mg days 1, 2, 5, 15, 17, 19, LEN 25 mg on days 1–21, and DEX 40 mg days 1, 8, 15 of 28 days cycle. The phase II study enrolled RRMM patients with a median of three prior therapies (range, 1–10); 81% were refractory to prior LEN [199]. ORR in the 27 evaluable patients was 41% (including two CR, four VGPR), with mPFS was 7.1 months. Protein expression levels of Cereblon, Ikaros, and Aiolos were similar between responders and non-responders, suggesting that PANO, rather than LEN, contributes to depth of response. Of the 22 LEN-refractory patients, 15 had at least MR (ORR of 36%), suggesting that PANO may enable recapturing of responses in refractory MM patients. The most common G3–4 AEs were hematologic: thrombocytopenia and neutropenia; non-hematologic G3–4 AEs included fatigue, infections and diarrhea. No dose interruption or reduction occurred for GI toxicity. PN was not reported, while only one patient discontinued therapy due to toxicity (asymptomatic QTc prolongation).

In a phase Ib study, Laubach et al. enrolled patients with RRMM to PANO 10–20 mg on days 1, 3, 5, 8, 10, and 12, LEN 15 mg on days 1–14 and BTZ 1 mg/m^2^ SQ on days 1, 4, 8, and 11 and DEX 20 mg on day of and after BTZ of 21 days cycle [200]. A total of 16 patients who received a median of four prior lines of therapy were enrolled. ORR was 36%. MTD of PANO was 10 mg. Grade 3–4 AEs in the 10 mg group included thrombocytopenia (18%), hypophosphatemia (18%), anemia, leukopenia, dyspnea, febrile neutropenia, hyperglycemia and lung infection (9% each). Shah et al. used the same 4-drug combination in patients (*n* = 52) with NDMM in a phase I/II trial at the dose of PANO 10 mg, LEN 25 mg, BTZ 1.3 mg/m^2^ SQ and DEX 20 mg, using the same schedule as the previous study [201]. LEN and PANO were continued as maintenance as tolerated. AHCT was allowed after cycle 4. After four cycles, ORR was 94% (67% ≥ VGPR). Half the patients were MRD negative. The regimen was well-tolerated; common G3–4 AEs included thrombocytopenia (36%), neutropenia (14%), and fatigue (12%). A phase I/II study of PANO with BTZ-THAL-DEX (PanoVTD) looking at the safety and efficacy of the regimen is underway (MUK-Six) [202]. In the phase I portion, patients with RRMM who had received 1–4 prior lines of therapy were treated with PanoVTD. The RP2D was determined to be PANO 20 mg on days 1, 3, 5, 8, 10, and 12; BTZ 1.3 mg/m^2^ SQ days 1 and 8; THAL 100 mg daily and DEX 20 mg on the days of and after BTZ in 3-weekly cycles. In the phase IIa portion, 46 patients were treated at the RP2D for up to 16 cycles followed by a year of PANO maintenance. AHCT was permitted after at least 6 cycles. Preliminary results showed an ORR of 91%. Treatment was overall well-tolerated: G3–4 AEs reported across both phases included neutropenia (25%), hypophosphatemia (19%), thrombocytopenia (14%), and diarrhea (10%). The incidence of AEs was lower than that reported in PANORAMA trials and that might be related to less frequent and SQ (rather than IV) dosing of BTZ in the MUK-Six trial [194].

### 6.3. Ricolinostat

To minimize toxicity seen with the use of pan-HDACi and to maintain efficacy, selective HDAC6 inhibitor, ricolinostat (ACY-1215) showed promise in pre-clinical testing and now clinical studies. Synergistic effects have been demonstrated with ACY-1215 and PIs [203]. Murine models of MM have demonstrated significant delay in tumor growth and significant prolongation of survival when treated with combination of ACY-1215 and BTZ [116,203]. As a single agent, ACY-1215 was well-tolerated in a phase I dose-escalation study [19,204]. ACY-1215 was combined with BTZ-DEX in a phase Ib trial by Vogl et al. in 57 RRMM patients (two-thirds were BTZ-refractory), who had received a median of 4 prior regimens (range, 2–13), using the following dosing schedule: ACY-1215 40–240 mg/d or 160 mg BID on days 1–5 and 8–12; BTZ 1–1.3 mg/m^2^ IV or SQ on days 1, 4, 8, and 11; and DEX 20 mg on the day of and the day after BTZ of 21 days cycle [205]. Treatment-emergent AEs were mainly G1–2, but common G3–4 AEs included thrombocytopenia, anemia, fatigue, hypokalemia, hyperglycemia, diarrhea, elevated liver enzymes and hyponatremia. AEs were more common with BID dosing. RP2D of ACY-1215 was 160 mg daily. Of 52 evaluable patients, ORR was 29% and CBR was 42%. Among BTZ-refractory patients, ORR was 13% and CBR was 31%. 

Yee et al. led a phase Ib dose-escalation study (ACE-MM-101) examining ACY-1215 in combination with LEN-DEX in patients with RRMM (*n* = 38) [206]. ORR in the 31 evaluable patients was 55% (1 sCR + 7 VGPR). The combination was well-tolerated. MTD was not reached. RP2D of ACY-1215 was established at 160 mg on days 1–21 of 28 days cycle. Raje et al. conducted a phase Ib/II trial (ACE-MM-102) investigating RCS 160 mg daily or BID on days 1–21 of 28 days cycle, in combination with POM-DEX in 73 RRMM patients [204]. Fatigue and diarrhea were the most common non-hematologic low-grade AEs (~40%); treatment-emergent G3–4 AEs were cytopenias and diarrhea. Though no DLT was observed, RP2D was set at 160 mg daily, as a result of diarrhea and fatigue with BID dosing. ORR was 42%, and was similar among dual-refractory (38%) and high-risk cytogenetics (55%) patients. The results from these early phase studies indicate clinical benefit of selective HDAC6 inhibition. The safety profile of ACY-1215 seems improved compared with PANO-based studies, but confirmation is warranted in phase III studies [19].

## 7. Conclusions

MM is a heterogeneous disease that is associated with complex genetic abnormalities and multiple signaling aberrations. Combining various drug classes with different mechanisms of action remains the cornerstone of myeloma treatment by targeting not only the MM cells but also the tumor microenvironment [207]. Over the last decade, proteasome has been validated as a novel and valuable target in MM [26]. Encouraged by the positive results of BTZ in MM, second generation PIs with different properties have been designed; these biological differences translate into improved efficacy and different toxicity. These emerging drugs have demonstrated promising anti-myeloma efficacy in relapsed and newly diagnosed MM and do not present high grades of PN, an AE that limits BTZ therapy. Even though it has been suggested that this side effect could be due to the broader range of proteasome subunits inhibition observed with BTZ, this does not seem to be the case with NPI-0052 that inhibits the three proteasome subunits but does not cause PN. It could be an off-target effect of BTZ [106] mediated by a proteasome-independent mechanism: BTZ inhibits several non-proteasomal targets in vitro and in vivo, which may play a role in its clinical AEs profile. Treatment with second-generation PIs has demonstrated partial cross-resistance with BTZ suggesting different mechanisms of resistance for these novel drugs. Therefore, one of the main avenues of research in the field of novel agent development is the understanding of resistance to these drugs. An in vivo model of acquired resistance dissecting the characteristics of the resistance and the main pathways involved may allow optimization of treatment sequence and help design therapeutic strategies using rational combinations so as to prevent the development of resistance [85].

HDACi have emerged as a unique treatment option for MM due to the aberrant epigenetic gene expression patterns and overproduction of misfolded proteins that are commonly present in MM cells. HDACi as monotherapy demonstrated only a modest clinical benefit, but rationally designed combination of PANO-BTZ-DEX in PANORAMA studies demonstrated a survival benefit in relapsed and/or refractory MM (particularly among those with at least two prior regimens including BTZ and IMiD). Efforts to improve the toxicity profile of this regimen by reducing and effective management of hematologic and GI AEs are necessary to optimize outcomes. HDAC6 inhibitor ACY-1215 demonstrated encouraging efficacy data and acceptable safety profile in early phase clinical trials [19]. Confirmatory studies are necessary to determine whether ACY-1215 has a better risk-benefit profile compared with PANO.

In conclusion, despite the availability of several new therapies in recent years, MM remains incurable with a high unmet need [19]. PIs are the backbone of the treatment of MM and combination therapies of the novel PIs with IMiDs, HDACi, and others are being explored. Some combinations have shown superior results and have been approved for treatment of RRMM, while others are in clinical trials. However, the current need is to develop drugs with novel mechanisms of action rather than newer drugs of the same class if the clinical efficacy, for example, of the novel PIs is not too far off from one another and the predicted toxicity does not conclusively guide specific treatment for certain unique patient-related or disease characteristics. HDACi meet the criteria for novel drug with different mechanism of activity and will serve as promising adjuncts in the treatment of MM. Future steps in the clinical development of PIs and HDACi include optimization of the schedule in different combinations and a better definition and characterization of their potentially synergistic antitumor activity, while counterbalancing their toxicity effectively. 

## Figures and Tables

**Table 1:** Characteristics of Second-generation Proteasome Inhibitors (PIs).

	Carfilzomib	Ixazomib	Oprozomib	Marizomib
**Active moiety**	Epoxyketone	Boronate	Epoxyketone	Beta-lactone
**Proteasome binding**	Irreversible	Reversible	Irreversible	Irreversible
**Proteasome inhibition**	Chymotrypsin-like >> caspase-and trypsin-like	Chymotrypsin-like > caspase- and trypsin-like	Chymotrypsin-like >> caspase-and trypsin-like	Chymotrypsin- and trypsin-like >> caspase-like
**Plasma half-life**	<30 min	18 min	<30 min	<5 min
**Mode of administration**	IV	Oral	Oral	IV
**Status**	FDA-approved with LEN-DEX as 2nd line and single-agent as 3rd line	FDA approved with LEN-DEX as 2nd line	Phase I	Phase I
**Unique off-target organ toxicity**	Cardiovascular	GI, myelosuppression, neurotoxicity	GI, myelosuppression	Central nervous system

Abbreviations: IV: Intravenous; FDA: Food and Drug Administration; LEN-DEX: lenalidomide-dexamethasone; GI: gastrointestinal.

**Table 2:** Landmark Clinical Trials of Novel PIs.

Investigator [Ref.] Study	Phase	Disease Status	#Prior Therapies Allowed	Treatment	Dosing Schedule of PI	N	Survival End-Point (Months or %)	ORR	≥VGPR Rate	Median Number of Prior Therapies	Prior Exposure	AEs
**Siegel DS [36]** **PX-171-003-A1**	II	RRMM	≥2	K (single-agent)	20/27 mg/m^2^ days 1, 2, 8, 9, 15, 16 q28days	266	mPFS 3.7, mOS 15.6	23.7%	5.5%	5	Prior BTZ 99.6% (73% refractory), prior AHCT 74%	NR
**Dimopoulos MA [37]** **ENDEAVOR**	III	RRMM	1–3	Kd vs. Vd	20/56 mg/m^2^ days 1, 2, 8, 9, 15, 16 q28days	929	mPFS 18.7 vs. 9.4	77% vs. 63%	54% vs. 29%	2	Previous BTZ 54% vs. 54%	SAE: 48% vs. 36%
**Stewart AK [38]** **ASPIRE**	III	RRMM	1–3	KRd vs. Rd	20/27 mg/m^2^ days 1, 2, 8, 9, 15, 16 q28days	792	mPFS 26.3 vs. 17.6	87% vs. 67%	70% vs. 40%	2	Previous BTZ 66% vs. 66%	SAE: 60% vs. 54%
**Berenson JR [39]** **CHAMPION**	I/II	RRMM	1–3	Kd	MTD 70 mg/m^2^ days 1, 8, 15 q28days	116	mPFS 12.6	77%	33%	1	BTZ-refract 55%	SAE: 35%
**Hajek R [40]** **FOCUS**	III	RRMM	≥3	K vs. d (+Cy)	20/27 mg/m^2^ days 1, 2, 8, 9, 15, 16 q28days	315	mPFS 3.7 vs. 3.3, mOS 10.2 vs. 10	19% vs. 11%			5	SAE: 59% vs. 51%
**Sonneveld P [41]**	II	NDMM	-	KTd	20/27–56 mg/m^2^ days 1, 2, 8,9, 15, 16 q28days	91	3-year PFS 72%	90%	68% after 4 cycles 89% after consolidat-ion	-	-	SAE: 40%
**Mikhael JR [42]** **CYKLONE**	I/II	NDMM	-	KCyTd	MTD of 20/36 mg/m^2^ IV d1,2,8,9,15,16 q28d	64	2-year PFS 76% and OS 96%	91%	69%			G ≥ 3 AE: 67%
**CLARION (unpublished)**	III	NDMM, AHCT-ineligible	-	KMP vs. VMP	CFZ 20/36 mg/m^2^ IV days 1, 2, 8, 9, 22, 23, 29, 30 q42days	955	mPFS 22.3 vs. 22.1	NR	NR	-	-	G ≥ 3 AEs: 75% vs. 76%
**Bringhen S [43]**	II	NDMM, AHCT-ineligible	-	KCyd	20/36 mg/m^2^ IV d1, 2, 8, 9, 15, 16 q28days	58	2-year PFS 76%, OS 87%	95%	71%	-	-	SAE: 28%
**Jakubowiak AJ [44]**	I/II	NDMM, AHCT-eligible and ineligible	-	KRd	20/20–36 mg/m^2^ IV d1, 2, 8, 9, 15, 16 q28days	53	3-year PFS 79.6%, OS 100%	100%	91%	-	-	NR
**Moreau P [45]** **TOURMALINE-MM1**	III	RRMM	1–3	IRd vs. Placebo-Rd	4 mg PO d1, 8, 15 q28d	722	mPFS 20.6 vs. 14.7	78% vs. 72%	48% vs. 39%	1; Prior auto 59% vs. 55%	Prior BTZ 69% vs. 69% Prior IMId 54% vs. 56%	SAE: 47% vs. 49%

Abbreviations: K: carfilzomib, V: bortezomib, Kd: carfilzomib-dexamethasone, Vd: bortezomib-dexamethasone, R: lenalidomide, d: low-dose dexamethasone, Cy: cyclophosphamide, M: melphalan, T: thalidomide, I: ixazomib, mPFS: median progression-free survival, mOS: median overall survival, ORR: overall response rate, VGPR: very good partial response, CR: complete response, RRMM: relapse/refractory multiple myeloma, NDMM: newly diagnosed multiple myeloma, AE: adverse events, SAE: serious adverse events, G: grade, MTD: maximum tolerated dose, AHCT: autologous hematopoietic cell transplantation, IMiD: immunomodulatory drug, NR: not reported.

**Table 3:** Landmark Clinical Trials of Histone Deacetylase Inhibitors.

Investigator [Ref.] Study	Phase	Disease Status	#Prior Therapies Allowed	Treatment	Dosing Schedule of the HDACi	N	Survival End-Point (Months or %)	ORR	≥VGPR Rate	Median Number of Prior Therapies	Prior Exposure	AEs
**Siegel DS [46] VANTAGE-095**	IIb	RRMM	≥2	Vorinostat-BTZ	Vorinostat 400 mg PO days 1–14 q21days	143	PFS 3.1, OS 11.2	11.3%	1% VGPR	2	BTZ-refractory 100%, IMiD-refractory 87%	SAE: 65%
**Dimopoulos M [47] VANTAGE-088**	III	RRMM	1–3	Vorinostat-Vd vs. placebo-Vd	Vorinostat 400 mg PO days 1–14 q21days	637	mPFS 7.6 vs. 6.8, mOS NA vs. 28	56% vs. 40%	CR 7.9% vs. 5.3%	2	Previous BTZ 25% vs. 23%	SAE: 41.3% vs. 43.1%
**San Miguel JF [48,49] PANORAMA 1**	III	RRMM	1–3	PanoVd vs. Placebo-Vd	Pano 20 mg PO days 1, 3, 5, 8, 10, 12 q21days	768	mPFS 12.1 vs. 8.1, mOS 40.3 vs. 35.8	61% vs. 55%	28% vs. 16%	2	Previous BTZ 51% vs. 52% Previous AHCT 56% vs. 59%	SAEs 60% vs. 42%

Abbreviations: R: lenalidomide, d: low-dose dexamethasone, M: melphalan, Pano: panobinostat, mPFS: median progression-free survival, mOS: median overall survival, ORR: overall response rate, VGPR: very good partial response, CR: complete response, RRMM: relapse/refractory multiple myeloma, NDMM: newly diagnosed multiple myeloma, AE: adverse events, SAE: serious adverse events, G: grade, MTD: maximum tolerated dose, Cy: cyclophosphamide, V: bortezomib, AHCT: autologous hematopoietic cell transplantation, PO: per os (by mouth).
